# LC-ESI QToF MS Non-Targeted Screening of Latex Extracts of *Euphorbia seguieriana* ssp. *seguieriana* Necker and *Euphorbia cyparissias* and Determination of Their Potential Anticancer Activity

**DOI:** 10.3390/plants12244181

**Published:** 2023-12-16

**Authors:** Milka Jadranin, Danica Savić, Ema Lupšić, Ana Podolski-Renić, Milica Pešić, Vele Tešević, Slobodan Milosavljević, Gordana Krstić

**Affiliations:** 1 University of Belgrade—Institute of Chemistry, Technology and Metallurgy, Department of Chemistry, Njegoševa 12, 11000 Belgrade, Serbia; danica.savic@ihtm.bg.ac.rs; 2 Department of Neurobiology, Institute for Biological Research “Siniša Stanković”—National Institute of the Republic of Serbia, University of Belgrade, Bulevar Despota Stefana 142, 11108 Belgrade, Serbia; ema.lupsic@ibiss.bg.ac.rs (E.L.); ana.podolski@ibiss.bg.ac.rs (A.P.-R.); camala@ibiss.bg.ac.rs (M.P.); 3 University of Belgrade—Faculty of Chemistry, Studentski trg 12–16, 11000 Belgrade, Serbia; vtesevic@chem.bg.ac.rs (V.T.); smilo@chem.bg.ac.rs (S.M.); 4 Serbian Academy of Science and Arts, Kneza Mihaila 35, 11000 Belgrade, Serbia

**Keywords:** *Euphorbiaceae*, non-targeted screening, jatrophanes, tiglianes, ingenanes, myrsinanes, premyrsinanes, P-gp function

## Abstract

*Euphorbia seguieriana* ssp. *seguieriana* Necker (ES) and *Euphorbia cyparissias* (EC) with a habitat in the Deliblato Sands were the subject of this examination. The latexes of these so far insufficiently investigated species of the *Euphorbia* genus are used in traditional medicine for the treatment of wounds and warts on the skin. To determine their chemical composition, non-targeted screening of the latexes’ chloroform extracts was performed using liquid chromatography coupled with quadrupole time-of-flight mass spectrometry employing an electrospray ionization source (LC-ESI QTOF MS). The analysis of the obtained results showed that the latexes of ES and EC represent rich sources of diterpenes, tentatively identified as jatrophanes, ingenanes, tiglianes, myrsinanes, premyrsinanes, and others. Examination of the anticancer activity of the ES and EC latex extracts showed that both extracts significantly inhibited the growth of the non-small cell lung carcinoma NCI-H460 and glioblastoma U87 cell lines as well as of their corresponding multi-drug resistant (MDR) cell lines, NCI-H460/R and U87-TxR. The obtained results also revealed that the ES and EC extracts inhibited the function of P-glycoprotein (P-gp) in MDR cancer cells, whose overexpression is one of the main mechanisms underlying MDR.

## 1. Introduction

Cancer is the second leading cause of mortality in the world. Many natural compounds such as anthracyclines (e.g., doxorubicin, DOX), vinca alkaloids (e.g., vincristine), podophyllotoxins (e.g., etoposide), and taxanes (e.g., taxol) are used for cancer therapy [1]. However, the main cause of unsuccessful cancer treatment is the development of multi-drug resistance (MDR) [2]. MDR is a phenomenon that indicates that cancer cells exhibit resistance to a number of chemotherapeutic agents with different structure and mode of action. One of the most relevant mechanisms underlying MDR is a decrease in the intracellular drug concentration due to the over-expression of the membrane transporter P-glycoprotein (P-gp) [3]. Thus, P-gp has become a significant target for overcoming MDR [4]. Many natural compounds from various sources possess the potential to modulate MDR [5]. Different metabolites isolated from *Euphorbia* ssp., besides antiproliferative and cytotoxic effects, showed potential to overcome MDR by P-gp inhibition [6].

The *Euphorbia* genus consists of over 2000 species of annual, biennial, or perennial flowering herbaceous plants, shrubs, trees, as well as cactus-like plants. Members of the genus are spread throughout the terrestrial part of the globe and grow in almost all habitats, in very different climatic conditions and soils of different quality. As a result of their great diversity in morphology, geographical distribution and habitat, *Euphorbia* species synthesize the most diverse metabolites, many of which are found in their milky latex. Latex is produced by all *Euphorbia* species in specialized laticifer cells and has a defensive role—it protects the plant from both mechanical injuries and injuries caused by herbivores (insects and mammals) [7] and various microorganisms. Latex was found to contain a broad range of specialized metabolites, different from those found in the corresponding plants, such as terpenoids, cardenolides, cerebrosides, alkaloids, and phenolics [8,9,10], which are partly responsible for their antibacterial, antifungal, anthelmintic, cytotoxic, and insect-repellent activities [11]. Latexes have also been recognized as reservoirs of defense-related proteins [7,12].

*Euphorbia seguieriana*, with three subspecies being recognized so far, i.e., *E. seguieriana* ssp. *hohenackeri* (Boiss.) Rech. fil., *E. seguieriana* ssp. *niciciana* (Borbás ex Novák) Rech. fil., and *E. seguieriana* ssp. *seguieriana* Necker, is one of the most widespread *Euphorbia* species inhabiting zonal and extrazonal steppes from Iberia to Central Asia (probably reaching China and Pakistan) [13]. It is a perennial herb that has a self-supporting growth form and reaches a height of up to 60 cm. Previous investigations mostly focused on the metabolites of the whole plant, and some bioactive diterpenoids with diverse structures, including abietane, myrsinane, a tetracarbocyclic diterpene related to myrsinane [14], hydroxymyrsinane, cyclomyrsinane, and lathyrane [15], as well as triterpene glycosides [16], phenolic compounds [17,18], flavonoids [19,20,21], proanthocyanidins [22], flavonoids, tannins, hydroxycinnamic acids [23], and alkaloids [24], were isolated and/or identified. Only a few investigations conducted on latex showed it contains ingenanes [25,26] and hydrolytic active proteins [27]. Although it is an irritant and a cocarcinogenic [25,26], the latex of *E. seguieriana* is used to treat wounds and warts on the skin [28].

The cypress spurge *E. cyparissias* L. is a hardy perennial, herbaceous plant growing in a wide range of habitats, from lowland areas to alpine locations. It is widely distributed in Europe (including in the Balkan Peninsula and Serbia) and Asia Minor, but it also occurs as an introduced plant in North America, Australia, Japan, and Hawaii. When the plant is cut, it secretes a white, bitter, and very spicy milk that causes inflammation and blisters on the skin and ocular inflammation [29]. The seeds are also pungent and poisonous, as is the whole plant. The roots of the plant were once used as a purgative. In people, the plant is still used for external treatments—removal of warts—while it is rarely used for its internal effects (inducing vomiting and purging). In previous investigations, ingenanes [30] and jatrophanes [31] were isolated from the roots and whole plant, respectively. In plant material other than latex, triterpenes [32,33,34,35], glycolipids [36], and flavonoids [37,38] were identified. For latex, only the identification of serine proteases [39] and invertase [40] has been reported.

The aim of the present work was to examine the chemical profiles of chloroform extracts of the latexes of *Euphorbia seguieriana* ssp. *segiueriana* Necker (ES) and *Euphorbia cyparissias* L. (EC) as sources of bioactive chemicals and whether these extracts can inhibit cancer cell growth and modulate P-gp function.

## 2. Results

### 2.1. Non-Targeted Screening of the Latex Chloroform Extracts Using Liquid Chromatography Coupled with Quadrupole Time-of-Flight Mass Spectrometry Employing an Electrospray Ionization Source

**Figure 1:**
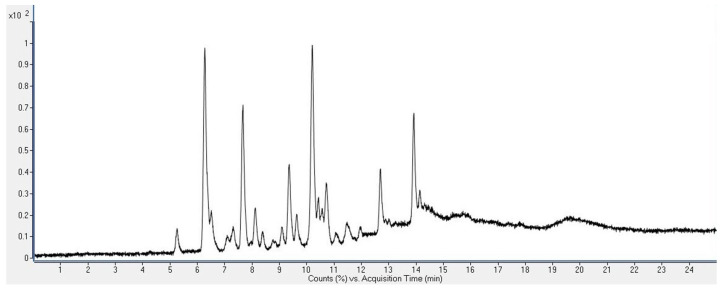
Total ion chromatogram of the chloroform extract of the latex of *E. seguieriana* ssp. *seguieriana* Necker (ES).

**Figure 2:**
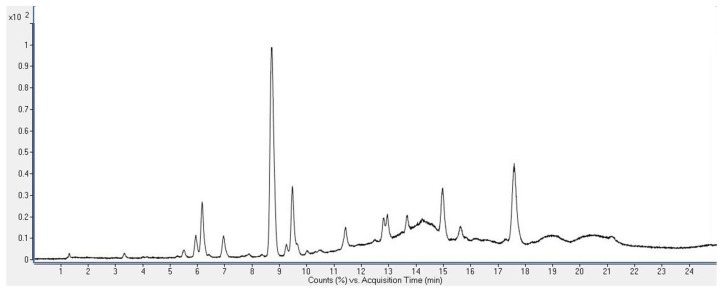
Total ion chromatogram of the chloroform extract of the latex of *E. cyparissias* (EC).

During the search for new sources of bioactive compounds, the chemical profiles of chloroform extracts of the latexes of ES and EC were investigated. For that purpose, liquid chromatography coupled with quadrupole time-of-flight mass spectrometry (LC-ESI QToF MS) in positive ion mode was employed. The total ion chromatograms of the chloroform extracts of the ES and EC latexes, obtained as a result of the analysis, are shown in Figure 1 and Figure 2, respectively.

The non-targeted screening of the ES extract allowed the detection of a total of 31 components, while a total of 49 metabolites were detected in the EC extract (Table 1 and Table 2, respectively). The chemical formulas of these components were determined based on mass accuracy, the number of double bond equivalents, the valency based on the nitrogen rule, and the isotopic pattern match of the suggested formula with the observed mass spectrum, as well chemical expertise. For a tentative identification of the metabolites, an extensive online literature search was conducted using the terms “*Euphorbia*, Euphorbiaceae” on SciFinder, an online database, for each proposed chemical formula. Also, the characteristic fragmentation pattern observed in the mass spectra of some of the detected metabolites allowed their closer class determination (Figures S1–S79, Supplementary Materials).

Diterpenoids were found to represent the most predominant chemical class in the examined extracts, but a smaller number of triterpene derivatives (in the EC extract) were also identified. LC-ESI QToF MS is more suitable for the analysis of diterpenes and other highly oxygenated molecules than for that of triterpene derivatives, which contain a small number of centers that can be ionized under soft ionization conditions. The weak ionization of triterpene derivatives can lead to the wrong conclusion that the presence of these compounds in the tested sample is small or negligible; however, our experience has shown that triterpenes are generally more abundant than expected, especially in non-polar extracts.

In soft ionization conditions, such as those used for recording the mass spectra of the components of the examined extracts, without additional collision energy, some compounds generate only quasimolecular ions, while other compounds spontaneously fragment (Figures S1–S79, Supplementary Materials), which indicates differences in the stability of their skeletons. Some diterpene esters produce fragment ions resulting from the neutral loss of water or acyl chains, which are not informative on the diterpene skeleton, but others, due to the presence of a different number of oxygenated groups, produce different characteristic fragment ions that could provide indications about the diterpene skeleton.

### 2.2. Examination of the Anticancer Activity of the ES and EC Latex Extracts

To evaluate the impact of the EC and ES extracts on the growth of human cell lines, including both normal and cancerous ones, we conducted an MTT assay. Our study included five different human cell lines, comprising two pairs of sensitive and MDR cancer cell lines (non-small cell lung carcinoma NCI-H460 and NCI-H460/R and glioblastoma U87 and U87-TxR cell lines) and normal human embryonic pulmonary fibroblasts (MRC-5). The results of the assay, which are outlined in Table 3, revealed that both EC and ES extracts had a significant impact on cancer cell growth, with IC50 values below 40 μg mL^−1^. However, we also observed that the efficacy of the extracts was affected by the presence of the MDR phenotype in NCI-H460/R cells. This was evidenced by a significant increase in the IC50 values for the MDR cells compared to those determined for the corresponding, sensitive NCI-H460 cells. It was also noted that this resistant profile was more pronounced in the case of the EC extract. Interestingly, both extracts were found to be almost equally effective in the sensitive U87 and MDR U87-TxR glioblastoma cells. Our analysis also indicated that the extracts were not selective towards cancer cells, as the normal MRC-5 cells exhibited lower IC50 values compared to those obtained for the cancer cells.

**Figure 3:**
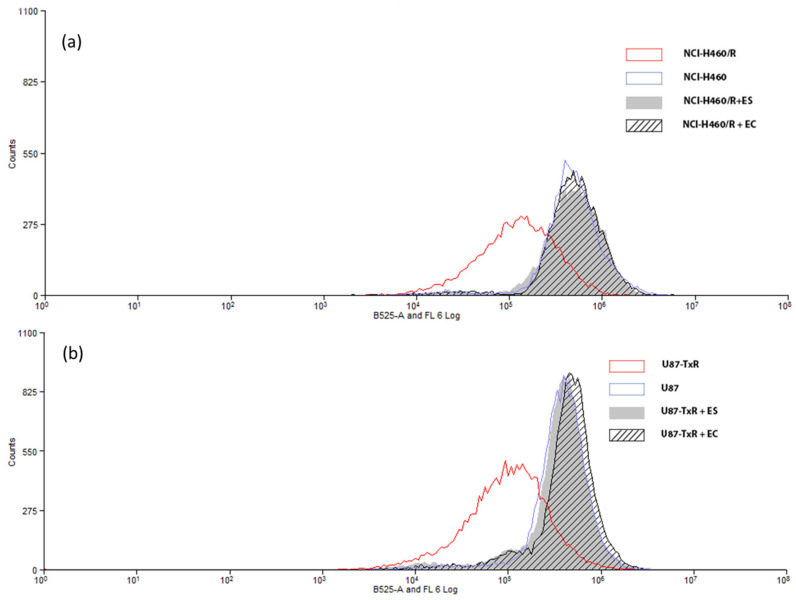
Flow cytometric profiles of Rho123 accumulation in NCI-H460/R (**a**) and U87-TxR (**b**) cells untreated and treated with 20 µg mL^−1^ of the ES and EC extracts. Sensitive NCI-H460 and U87 cells were used as a positive control for Rho 123 accumulation. Two independent experiments were performed (a minimum of 10,000 events were collected for each experimental sample).

To investigate whether the ES and EC extracts affect the function of the P-gp pump in MDR cancer cells, the intracellular accumulation of the P-gp substrate Rho 123 was analyzed by flow cytometry after a 30 min treatment (Figure 3). Both extracts were applied at 20 µg mL^−1^. As shown by a marked increase in Rho 123 intracellular accumulation, the ES and EC extracts significantly inhibited P-gp function in both MDR cancer cell lines.

## 3. Discussion

### 3.1. Non-Targeted Screening of the Latex Chloroform Extracts Using Liquid Chromatography Coupled with Quadrupole Time-of-Flight Mass Spectrometry Employing an Electrospray Ionization Source

The soft ionization conditions applied for the LC-ESI QToF MS analysis in positive ion mode allowed, based on the precisely measured mass of molecular ions, the determination of the molecular formula of the components present in the tested latex chloroform extracts of ES and EC, while an extensive online literature search using the terms “*Euphorbia*, Euphorbiaceae” in SciFinder, an online database, and the characteristic fragmentation pattern observed in the corresponding mass spectra enabled the tentative identification and chemical class determination of the majority of the components (Table 1 and Table 2, Figures S1–S79, Supplementary Materials). In total, twenty components could not be tentatively identified in this way, seven of which were in the ES extract (**3**: C_40_H_47_NO_13_, t*_R_* = 6.49 min, **5**: C_35_H_40_O_11_, t*_R_* = 7.07 min, **6**: C_44_H_47_NO_12_, t*_R_* = 7.11 min, **19**: C_40_H_47_NO_11_, t*_R_* = 10.21 min, **21**: C_42_H_49_NO_11_, t*_R_* = 10.57 min, **25**: C_40_H_46_O_11_, t*_R_* = 11.95 min, and **27**: C_43_H_50_O_11_, t*_R_* = 12.89 min) and thirteen in the EC extract (**39**: C_40_H_48_O_13_, t*_R_* = 5.97 min, **41**: C_36_H_48_O_12_, t*_R_* = 6.42 min, **49**: C_45_H_46_O_13_, t*_R_* = 9.36 min, **52**: C_47_H_50_O_14_, t*_R_* = 9.70 min, **57**: C_42_H_46_O_12_, t*_R_* = 10.43 min, **59**: C_36_H_46_O_10_, t*_R_* = 10.49 min, **60**: C_40_H_48_O_11_, t*_R_* = 10.54 min, **61**: C_45_H_46_O_12_, t*_R_* = 11.33 min, **63**: C_31_H_52_O_5_, t*_R_* = 11.64 min, **64**: C_45_H_46_O_13_, t*_R_* = 12.51 min, **70**: C_40_H_58_O_8_, t*_R_* = 14.59 min, **75**: C_39_H_72_O_7_, t*_R_* = 17.25 min, and **79**: C_39_H_54_O_7_, t*_R_* = 18.25 min), suggesting the presence of so far undescribed compounds in the Euphorbiacea family. In addition to these, also the compound with molecular formula C_22_H_42_O_4_ (**31** or **78**, t*_R_* = 17.78 min), detected in both extracts, could not be identified, although chemical expertise suggested it to be a diester of dicarboxylic acid.

Diterpenoids were found to represent the most predominant chemical class in the examined extracts, but triterpene derivatives (in the EC extract) were also identified.

The compound with molecular formula C_39_H_45_NO_12_ was detected in both extracts, but at different retention times in the chromatograms (**1**: t*_R_* = 5.26 min in the ES extract, and **43**: t*_R_* = 7.76 min in the EC extract), indicating the existence of two different metabolites. Almost half of the detected metabolites in the ES extract appeared to contain nitrogen, while in the EC extract, only three metabolites, including amino acid **32** (C_7_H_15_NO_2_ at t*_R_* = 1.31 min), were shown to contain nitrogen, thus indicating the presence or absence of a nicotinoyl ester group in their structures. Only three metabolites detected in the ES extract showed the same molecular formulas as myrsinanes isolated and characterized in previous research on *E. seguieriana* [14]; those metabolites are **4**: C_39_H_43_NO_11_, t*_R_* = 6.52 min, **9**: C_35_H_43_NO_11_, t*_R_* = 7.28 min, and **17**: C_40_H_45_NO_11_, t*_R_* = 9.36 min. Ingenanes contained in the latex of *E. seguierina* [25,26] were not detected in our study in the ES extract. In the EC extract, only four metabolites, i.e., three ingenanes (**66**: C_38_H_58_O_10_, t*_R_* = 12.96 min, **71**: C_36_H_56_O_8_, t*_R_* = 14.98 min, and **77**: C_38_H_60_O_8_, t*_R_* = 17.59 min) and one triterpene (**74**: C_30_H_48_O_2_, t*_R_* = 16.18 min), showed the same molecular formulas as those of compounds isolated and characterized in previous research on *E. cyparissias* [30,34]. However, two jatrophane diterpenes (cyparissins A and B) with molecular formula C_38_H_42_O_12_, previously isolated from *E. cyparissias* [31], were not detected in the examined EC extract. These findings indicate the ecological importance of the collection site.

A literature survey showed that compounds **15**, **18**, **20**, **22**–**24**, and **26** are premirsinane-, lathyrane-, or jatrophane-type diterpene esters [48,52,53,59,61,62,63,64,65,66]. In the experimental mass spectra of all these components, the fragment ions 313, 295, and 267, characteristic of ingenane esters/deoxyphorbol esters (IEs/dPEs), could be observed, once more providing evidence that other types of diterpene esters can also produce IE/dPE-like fragmentation [160]. This ambiguity did not allow the identification of compound **62**, for which the mass spectrum fragment ions 313, 295, and 267 were observed, and which could have an ingenane or lathyrane skeleton [122,123,124].

Fragment ions 311, 293, and 265, characteristic of phorbol esters (PEs) [160] and some ingenanes [161], could be observed in the mass spectrum of compound **28**, while, according to the literature data, the only compound with molecular formula C_38_H_50_O_9_ so far identified in the genus *Euphorbia* belong to the dPE type of diterpenes [68,69]. Similarly, the same fragment ions occurred in the mass spectrum of compound **67**, while the only compound with molecular formula C_37_H_50_O_8_ so far identified in the Euphorbiacea family belong to the daphnane type of diterpenes [129].

In the ES extract, four pairs of isobaric compounds were detected: two compounds with molecular formula C_36_H_46_O_12_—**7** at t*_R_* = 7.12 and **13** at t*_R_* = 8.39 min—and two compounds with molecular formula C_41_H_48_O_12_—**18** at t*_R_* = 9.62 and **22** at t*_R_* = 10.69 min—while only one *Euphorbia*/Euphorbiaceae premyrsinane with a corresponding molecular formula has been identified from each pair so far [44,51,52,53,61], in addition to two compounds with molecular formula C_36_H_48_O_12_—**10** at t*_R_* = 7.33 and **14** at t*_R_* = 8.77 min—corresponding to two known premyrsinanes [46,47,57], and two compounds with molecular formula C_36_H_50_O_8_—**29** at t*_R_* = 13.92 min and **30** at t*_R_* = 14.13 min—whose mass spectra showed fragment ions corresponding to the loss of a water molecule, as well as fragment ions 313, 295, and 267. The only compound with the same molecular formula so far identified in the genus *Euphorbia* belongs to the PE type of diterpene esters [70,71,72,73].

In the EC extract, five pairs of isobaric compounds were detected: two compounds with molecular formula C_38_H_44_O_12_—**38** at t*_R_* = 5.94 min and **40** at t*_R_* = 6.17 min—in whose mass spectra, fragment ions corresponding to the loss of a water molecule and a benzoic acid molecule could be observed, as occurs with four known jatrophans with the same formula [77,102,103]; two compounds with molecular formula C_38_H_42_O_11_—**44** at t*_R_* = 7.88 min and **47** at t*_R_* = 8.70 min—with the observation, in the mass spectrum of the latter, of a fragment ion characteristic of the loss of benzoic acid, which is a substituent in three ingols [88,101,105] and one jatrophane [86]; two compounds with molecular formula C_40_H_44_O_12_—**48** at t*_R_* = 9.26 min and **50** at t*_R_* = 9.47 min—in whose mass spectra, fragment ions corresponding to the loss of a benzoic acid molecule, present as a substituent in two known ingols [88] and one known jatrophane [86,93,107], could be observed; two compounds with molecular formula C_38_H_48_O_12_—**54** at t*_R_* = 10.19 min and **56** at t*_R_* = 10.33 min—corresponding to two known jatropahanes [114,115] and one known myrsinane [57]; and two compounds with molecular formula C_39_H_54_O_8_—**69** at t*_R_* = 14.22 min and **72** at t*_R_* = 15.63 min—corresponding to two known ingenanes [138].

Fragment ions 313, 295, and 267 could be observed in the mass spectra of compounds **65** and **66**, while fragment ions 311, 293, and 265 could be observed in the mass spectra of compounds **71**–**73**. All these compounds, according to the literature data, have an ingenane or tigliane skeleton [30,113,125,126,127,128,138,139,140,141].

Compounds **33** [76,77,78], **41**, and **49** produce fragment ions corresponding to the loss of a water molecule, and compounds **35**, **37**, **47**, **48**, **50**, and **51** produced fragment ions corresponding to the loss of benzoic acid, which agrees with the literature data [60,83,84,85,86,87,88,89,90,91,92,93,94,95,96,97,98,99,100,101,105,107,108,109], while in the mass spectra of compounds **38**, **40**, and **72**, fragment ions corresponding to the loss of a water molecule and benzoic acid could be observed, which also agrees with the literature data [77,102,103,138]. In the mass spectrum of compound **77**, fragment ions corresponding to the loss of CO, C_5_H_11_OH, and two molecules of water could be observed, in addition to fragment ions 311, 293, and 265, which agrees with the literature data [30,128,139,156,157,158].

Compounds **27** and **61**, as well compounds **64** and **70**, so far undescribed in the genus *Euphorbia* and Euphorbiaceae family, produced fragment ions 313, 295, and 267, characteristic of the IE/dPE type of diterpenes [160], and fragment ions 311, 293, and 265, characteristic of the PE type of diterpenes and of some ingenanes [160,161].

The incomplete identification of the components present in the investigated extracts is the main drawback of this study and reflects the limitations of LC-ESI QToF MS in the annotation of compounds such as diterpene esters. For the complete identification of the components present in the examined extracts, the isolation and characterization of the compounds are required.

### 3.2. Examination of the Anticancer Activity of the ES and EC Latex Extracts

As shown by the analysis of the data available in the literature on the biological activities of the classes of molecules detected in the ES and EC extracts by LC-ESI QToF MS, the results obtained in this research confirmed the literature data. Our research indicated that both extracts of EC and ES have the potential to inhibit the growth of cancer cells. However, their effectiveness may be reduced in the case of MDR cancer cells, especially that of the EC extract. We discovered that both extracts could increase the accumulation of the P-gp substrate Rho123, which suggests that some compounds present in the extracts may be P-gp substrates that can also competitively inhibit P-gp activity. This is likely the reason for the decreased efficacy of the extracts in MDR cancer cells, such as MDR non-small cell carcinoma cells. Additionally, some components of the extracts are toxic to normal cells, which raises concerns about their use as anticancer agents. Nevertheless, the presence of different bioactive compounds suggests that some of them may be selective against cancer cells, while others are not. Therefore, further testing of isolated compounds is necessary to identify the best candidates as anticancer agents and lead compounds.

The potential of ES and EC to inhibit P-gp could be attributed to jatrophane derivatives identified in both extracts. In fact, the largest number of identified metabolites in the EC extract belong to the jatrophane class, while in the ES extract, jatrophane derivatives appeared to be the second most abundant metabolites. Our previous study demonstrated that jatrophane diterpenoids isolated from the latex of *Euphorbia dendroides* were able to modify P-gp function in three different human MDR cancer cell lines, i.e., non-small cell lung carcinoma, colorectal carcinoma, and glioblastoma cell lines [162]. Further study also showed that jatrophane diterpenoids isolated from the latex of *Euphorbia nicaeensis* collected in Serbia possessed P-gp-inhibiting activity in two MDR cancer cells of different origin [58]. Also, other compounds detected in the EC and ES extracts, such as lathyranes, are known as potent P-glycoprotein inhibitors in the treatment of multidrug-resistant (MDR) cancers [88,163,164]. Jo et al. determined the anti-proliferative potential of daphnane derivatives in lung cancer cells, finding IC_50_ values in the nM range [165]. At the same time, the tested compounds showed selectivity towards carcinoma cells compared to MRC-5 cells [165]. The difference in the IC_50_ values of the examined extracts for the NCI-H640 cell line and the stronger anti-cancer activity of the EC extract compared to the ES extract can be explained by the potential presence of daphnane diterpenes in the EC extract. Strong inhibitory activity against the human glioblastoma cell line U87 was demonstrated for triterpene lanostane derivatives isolated from the fungus *Naematoloma fasciculare* [166]. Lanostane derivatives are frequent metabolites in the *Euphorbia* genus; so, additional experiments and compound isolation are necessary to determine whether lanostane derivatives are responsible for the inhibitory activity of the extracts in the U87 cell line [134,167].

## 4. Materials and Methods

### 4.1. Plant Materials

The latexes of ES (N 44°59′07.0″, E 21°01′20.4″) and EC (N 45°00′00.5″, E 21°01′11.5″) were collected from wild stock in Deliblato Sands (Serbia) in May 2022. The plants were identified by Professor Marjan Niketić, Serbian Academy of Sciences and Arts, Belgrade. Voucher specimens (BEOU17883 and BEOU17893, respectively) were deposited at the Herbarium of the Natural History Museum—Belgrade (Serbia).

### 4.2. Chemicals

Chloroform (for HPLC, >99.8%, amylene-stabilized, Sigma-Aldrich, Saint-Quentin-Fallavier, France), dichlorometane (for HPLC, isocratic grade, stabilized with ethanol, Carlo Erba, France), acetonitrile (LiChrosolv^®^, hypergrade for LC-MS, Merck, Darmstadt, Germany), and deionized water (18.2 MΩcm^−1^, Barnstead™ Smart2Pure™ Water Purification System, Thermo Scientific™, Waltham, MA, USA) were used for sample extraction, dissolution, and preparation of the mobile phases for the LC-ESI QTOF MS analysis. Ammonium formate (puriss. *p.a.*, eluent additive for LC-MS, Fluka, Honeywell International, Inc., Charlotte, NC, USA) and formic acid (eluent additive for LC-MS, Fluka Analytical) were used for the preparation of eluent additives for LC-ESI QTOF MS.

### 4.3. Sample Preparation and Liquid Chromatography-Electrospray Quadrupole Time-of-Flight Mass Spectrometry (LC-ESI QTOF MS) Measurements

Two hundred microliters of each ES and EC latex were suspended in 700 µL of chloroform (to remove macromolecular substances such as proteins and polysaccharides), followed by 5 min of shaking and separation of the chloroform layer. After evaporation of the solvent under a mild nitrogen stream, the solid residue was dissolved in 1 mL of a mixture of dichloromethane and acetonitrile (1:5, *v*/*v*), filtered through Captiva RC 0.45 mm filters (Agilent Technologies, Waldbronn, Germany), and analyzed by liquid chromatography-electrospray quadrupole time-of-flight mass spectrometry (LC-ESI QTOF MS), as described below. For the untargeted analysis, the prepared samples were injected into the analyzing system, including a liquid chromatograph (1290 Infinity LC system; Agilent Technologies, Waldbronn, Germany) with a quaternary pump, a column oven, and an autosampler, connected to a quadrupole time-of-flight mass detector (6550 iFunnel Q-TOF MS, Agilent Technologies; Santa Clara, CA, USA) equipped with a dual-spray Agilent Jet Stream (AJS) electrospray ion source [168,169]. In this case, the separation of the compounds was performed using a Zorbax Eclipse XDB-C18 RRHT column (100 × 4.6 mm, 1.8 μm, Agilent Technologies). The mobile phase was composed of solvents A (water containing both 0.1% formic acid and 5 mM ammonium formate) and B (ACN containing 0.1% formic acid). The following gradient program was used: 0–2 min 60% B, 2–12 min 60–95% B, 12–18 min 95% B, and 5 min 60% B. The mobile phase flow rate was 0.60 mL min^−1^, the column temperature was 50 °C, and the injection volume of the samples was 0.1 μL. After separation, the compounds were analyzed using a mass detector. Positive ion mode was used, and the instrument was operated in accurate TOF/MS scanning mode in the *m*/*z* range of 100–2000, under the following conditions: capillary voltage, 3500 V, fragmentor voltage, 70 V, nozzle voltage, 1000 V, skimmer 1, 65 V, octupole RF peak, 750 V, desolvation gas (nitrogen) temperature, 200 °C, desolvation gas (nitrogen) flow, 14 L min^−1^, nebulizer pressure, 35 psi, sheath gas (nitrogen) temperature, 350 °C, and sheath gas (nitrogen) flow, 11 L min^−1^. A calibrating solution containing internal reference masses at *m*/*z* 121.0508 and 922.0098 was used in conjunction with an automated calibration delivery system to obtain accurate mass measurements for each peak in the total ion chromatogram. A personal computer system running Agilent MassHunter software (revisions B.06.01 and B.07.00) was used for data acquisition and processing. Extraction of the raw data (d) using both the find-by-molecular-feature (MFE) and the find-by-formula algorithms (FBF) in Agilent MassHunter Qual. software (revision B.07.00) allowed the detection of compounds in the tested samples.

### 4.4. Drugs

The extracts of EC and ES were kept as 20 mg mL^−1^ stocks in 100% ethanol at −20 °C. Working solutions were prepared in deionized water.

### 4.5. Cells and Cell Culture

The NCI-H460 and U87 cell lines were bought from the American Type Culture Collection, Manassas, VA, USA, while the MRC-5 cell line was obtained from the European Collection of Authenticated Cell Cultures, Salisbury, UK. NCI-H460/R and U87-TxR cells were created by exposing NCI-H460 and U87 cells to increasing concentrations of doxorubicin and paclitaxel, respectively, in order to kill sensitive cells and obtain cells resistant to many structurally and functionally unrelated drugs [170,171]. NCI-H460 and NCI-H460/R cells were cultured in RPMI 1640 medium supplemented with 10% fetal bovine serum, L-glutamine, and an antibiotic–antimycotic mixture, U87 and U87-TxR cells were cultured in MEM medium supplemented with 10% fetal bovine serum, L-glutamine, antibiotics, and non-essential amino acids, and MRC-5 cells were cultured in DMEM supplemented with 10% fetal bovine serum, 4 g L^−1^ of glucose, L-glutamine, and an antibiotic–antimycotic mixture. The cells were sub-cultured twice a week and seeded into fresh medium at a density of 8000 cells cm^−2^ (NCI-H460 and NCI-H460/R cells) or 16,000 cells cm^−2^ (U87, U87-TxR, and MRC-5 cells).

### 4.6. Cell Viability Assay

To determine cell viability, we employed the MTT assay, which is based on the ability of active mitochondria in living cells to reduce 3-(4,5-dimethyl-2-thiazolyl)-2,5-diphenyl-2H-tetrazolium bromide into a formazan dye [172]. We initially seeded the cells in 96-well tissue culture plates, seeding 2000 cells/well for NCI-H460 and NCI-H460/R cells and 4000 cells/well for U87, U87-TxR, and MRC-5 cells, and incubated them overnight in appropriate medium. We then treated the cells with varying concentrations of the EC and ES extracts—1, 5, 10, 25, and 50 µg mL^−1^—for 72 h.

Following the treatment, we added MTT to each well at a final concentration of 0.2 mg mL^−1^ and left it for 4 h. We subsequently dissolved the formazan product in dimethyl sulfoxide and measured the absorbance at 570 nm using an automatic microplate reader (Multiskan Sky from Thermo Scientific, Waltham, MA, USA). Using non-linear regression analysis in GraphPad Prism 8 software, San Diego, CA, USA, we calculated the IC50 values, which represent the concentration of each extract that inhibited cell growth by 50%.

### 4.7. Rhodamine 123 Accumulation Assay

We conducted an investigation using flow cytometry to examine the function of P-gp, a protein that transports substances out of cells. Specifically, we wanted to see how the EC and ES extracts affected the accumulation of the P-gp substrate rhodamine 123 (Rho123) [173] in two types of P-gp-overexpressing cells (NCI-H460/R and U87-TxR) and compared the results with those from control cells (NCI-H460 and U87). To carry out the experiment, we grew all cell lines to 80% confluence in 25 cm^2^ flasks, collected the cells, and put them in a solution containing Rho123 (2.5 µmol L*^−^*^1^). We immediately treated the MDR cells with the EC and ES extracts (20 µg mL*^−^*^1^, the average IC50 calculated for all tested cancer cell lines) and incubated them at 37 °C in 5% CO*_2_* for 30 min. After the accumulation period, we washed the samples twice, collected the cells, and analyzed them using a CytoFLEX flow cytometer (Beckman Coulter, IN, USA). The orange fluorescence of Rho123 was measured on fluorescence channel 1 (FL1) at 525 nm. We tested at least 20,000 events for each sample, and the mean fluorescence intensities were analyzed using Summit v4.3 software (Dako Colorado Inc., Fort Collins, CO, USA). We analyzed the mean ± SEM values from three independent experiments using GraphPad Prism 8 (San Diego, CA, USA) and used Sidak’s multiple comparison test for two-way ANOVA for the statistical analysis.

## 5. Conclusions

The selected plant species proved to be a rich source of biologically active compounds, primarily from the class of diterpenes. The small number of references on the chemical composition of these plant species, as well as the very limited number of ambiguous literature data on the mass spectra of *Euphorbia* diterpenes indicate the necessity of a detailed examination of the numerous compounds of this class that we detected. From the available literature data, it is known that, from *E. cyparissias*, two jatrophane diterpenes (cyparissins A and B) with the molecular formula C_38_H_42_O_12_ were isolated, which were not detected in the examined extract, which further indicates the need to investigate this plant species in more detail because habitat conditions can also significantly affect the metabolites synthesized by the plant.

Another important result from this study is the finding that the extracts obtained from *E. seguieriana* and *E. cyparissias* showed the ability to inhibit P-gp function. The results of our study may contribute to the development of more effective cancer treatments in the future.

## Figures and Tables

**Table 1:** Tentative identification of the components of the chloroform extract of the latex of *E. seguieriana* ssp. *seguieriana* Necker (ES) by LC-QToF MS according to the literature data available in SciFinder, an online database.

No.	RT (min)	Ion Species	*m*/*z* Measured	Molecular Mass Measured	Proposed Formula	Molecular Mass Calculated	Diff. (ppm)	Compound (CAS No.) [Ref.]	Class
**1**	5.26	[M+H]^+^ [M+Na]^+^ [M+K]^+^	720.3018 742.2834 758.2570	719.2944	C_39_H_45_NO_12_	719.2942	0.29	2595240-63-2 [41] 2595235-60-0 [41] 2595235-05-3 [41] 2595233-36-4 [41] 777896-12-5 [42] 2408424-43-9 [43] 2342577-75-5 [44]	Jatrophane Jatrophane Jatrophane Jatrophane Jatrophane Premyrsinane Premyrsinane
**2**	6.27	[M+H]^+^ [M+Na]^+^ [M+K]^+^	734.3173 756.2992 772.2729	733.3155	C_40_H_47_NO_12_	733.3098	2.28	1615711-25-5 [45] 1380589-96-7 [44,46,47] 2112824-86-7 [48] 1980015-12-0 [49] 1778734-87-4 [50]	Tetrahydroingenoid Premyrsinane Premyrsinane Premyrsinane Premyrsinane
3 *	6.49	[M+H]^+^ [M+Na]^+^ [M+K]^+^	750.3124 772.2927 788.2673	749.3039	C_40_H_47_NO_13_	749.3047	−1.11	/	/
**4**	6.52	[M+H]^+^ [M+NH_4_]^+^ [M+Na]^+^	717.3019 734.3173 739.2826	716.2928	C_39_H_44_N_2_O_11_	716.2945	−2.35	171864-09-8 [14,46]	Myrsinane
5 *	7.07	[M+H]^+^ [M+NH_4_]^+^	637.2615 654.2910	636.2570	C_35_H_40_O_11_	636.2571	−0.04	/	/
6 *	7.11	[M+H]^+^ [M+Na]^+^ [M+K]^+^	782.3168 804.2986 820.2721	781.3094	C_44_H_47_NO_12_	781.3098	−0.54	/	/
7 *	7.12	[M+H]^+^ [M+Na]^+^ [M+K]^+^	671.3060 693.2894 709.2617	670.2991	C_36_H_46_O_12_	670.2989	0.26	247099-10-1 [44,51,52,53]	Premyrsinane
8 *	7.24	[M+H]^+^ [M+Na]^+^ [M+K]^+^	790.3072 812.2888 828.2625	789.2997	C_42_H_47_NO_14_	789.2997	0.12	1380590-01-1 [46]	Cyclomyrsinane
9 *	7.28	[M+H]^+^ [M+NH_4_]^+^	654.2911 671.3058	653.2837	C_35_H_43_NO_11_	653.2836	0.14	171864-14-5 [14,54,55] 1799735-20-8 [56]	Myrsinane Myrsinane
**10**	7.33	[M+H]^+^ [M+Na]^+^ [M+K]^+^	748.3331 770.3146 786.2883	747.3256	C_41_H_49_NO_12_	747.3255	0.17	1928726-37-7 [47,57] 1380589-97-8 [46,47]	Premyrsinane Premyrsinane
**11**	7.67	[M+H]^+^ [M+Na]^+^ [M+K]^+^	734.3175 756.2991 772.2719	733.3101	C_40_H_47_NO_12_	733.3098	0.40	1615711-25-5 [45] 1380589-96-7 [44,46,47] 2112824-86-7 [48] 1980015-12-0 [49] 1778734-87-4 [50]	Ingenoid Premyrsinane Premyrsinane Premyrsinane Premyrsinane
**12**	8.12	[M+H]^+^ [M+Na]^+^	656.3064 678.2878	655.2991	C_35_H_45_NO_11_	655.2993	−0.32	2222920-06-9 [58]	Jatrophane
**13**	8.39	[M+H]^+^ [M+NH_4_]^+^ [M+Na]^+^ [M+K]^+^ [2M+Na]^+^	671.3063 688.3329 693.2884 709.2621 1363.5871	670.2990	C_36_H_46_O_12_	670.2989	0.15	247099-10-1 [44,51,52,53]	Premyrsinane
14 *	8.77	[M+H]^+^ [M+Na]^+^ [M+K]^+^	748.3332 770.3149 786.2886	747.3257	C_41_H_49_NO_12_	747.3255	0.37	1928726-37-7 [47,57] 1380589-97-8 [46,47]	Premyrsinane Premyrsinane
**15**	9.08	[M+H]^+^ [M+NH_4_]^+^ [M+Na]^+^ [M+K]^+^ [2M+Na]^+^ [2M+K]^+^	629.2948 646.3220 651.2777 667.2511 1279.5629 1295.5367	628.2882	C_34_H_44_O_11_	628.2884	−0.23	2112824-87-8 [48] 1801541-77-4 [53]	Premyrsinane Premyrsinane
16 *	9.34	[M+H]^+^ [M+Na]^+^ [M+K]^+^	704.3074 726.2892 742.2619	703.2999	C_39_H_45_NO_11_	703.2993	0.87	1529776-07-5 [59] 777896-21-6 [42]	Premyrsinane Jatrophane
**17**	9.36	[M+H]^+^ [M+NH_4_]^+^ [M+Na]^+^	716.3066 733.3201 738.2873	715.2993	C_40_H_45_NO_11_	715.2993	0.07	171864-12-3 [14,46,52,60]	Myrsinane
**18**	9.62	[M+H]^+^ [M+Na]^+^ [M+K]^+^	733.3221 755.3040 771.2779	732.3147	C_41_H_48_O_12_	732.3146	0.15	2674753-70-7 [61]	Premyrsinane
**19**	10.21	[M+H]^+^ [M+Na]^+^ [M+K]^+^ [2M+Na]^+^	718.3225 740.3040 756.2777 1457.6217	717.3151	C_40_H_47_NO_11_	717.3149	0.31	/	/
**20**	10.43	[M+H]^+^ [M+NH_4_]^+^ [M+Na]^+^ [M+K]^+^ [2M+Na]^+^	591.2949 608.3219 613.2775 629.2511 1203.5657	590.2881	C_35_H_42_O_8_	590.2880	0.30	1809418-89-0 [62,63]	Lathyrane
**21**	10.57	[M+H]^+^ [M+Na]^+^ [M+K]^+^	744.3382 766.3201 782.2932	743.3308	C_42_H_49_NO_11_	743.3306	0.29	/	/
**22**	10.69	[M+H]^+^ [M+NH_4_]^+^ [M+Na]^+^ [M+K]^+^	733.3217 750.3487 755.3042 771.2778	732.3148	C_41_H_48_O_12_	732.3146	0.35	2674753-70-7 [61]	Premyrsinane
**23**	10.73	[M+NH_4_]^+^ [M+Na]^+^ [M+K]^+^ [2M+Na]^+^	672.3380 677.2964 693.2670 1331.5980	654.3057	C_36_H_46_O_11_	654.3040	2.64	1335200-98-0 [52] 1333481-71-2 [64,65] 173967-58-3 [66]	Premyrsinane Premyrsinane Premyrsinane
24 *	11.06	[M+NH_4_]^+^ [M+Na]^+^ [M+K]^+^	698.3534 703.3089 719.2825	680.3197	C_38_H_48_O_11_	680.3197	0.09	1946844-21-8 [67]	Jatrophane
25 *	11.95	[M+NH_4_]^+^ [M+Na]^+^ [M+K]^+^	720.3378 725.2934 741.2664	702.3040	C_40_H_46_O_11_	702.3040	0.00	/	/
**26**	12.70	[M+NH_4_]^+^ [M+Na]^+^ [M+K]^+^ [2M+Na]^+^	734.3536 739.3092 755.2826 1455.6305	716.3199	C_41_H_48_O_11_	716.3197	0.29	1529776-06-4 [59]	Premyrsinane
27 *	12.89	[M+NH_4_]^+^ [M+Na]^+^ [M+K]^+^	760.3690 765.3247 781.2977	742.3353	C_43_H_50_O_11_	742.3353	−0.05	/	/
28 *	13.01	[M+NH_4_]^+^ [M+Na]^+^ [M+K]^+^	668.3791 673.3347 689.3083	650.3454	C_38_H_50_O_9_	650.3455	−0.14	72826-62-1 [68,69]	Tigliane
**29**	13.92	[M+H]^+^ [M+NH_4_]^+^ [M+Na]^+^ [M+K]^+^ [2M+NH_4_]^+^ [2M+Na]^+^	611.3574 628.3842 633.3397 649.3132 1238.7358 1243.6920	610.3505	C_36_H_50_O_8_	610.3506	-0.10	57672-63-6 [70,71,72,73]	Tigliane
**30**	14.13	[M+H]^+^ [M+NH_4_]^+^ [M+Na]^+^ [M+K]^+^ [2M+Na]^+^	611.3575 628.3843 633.3399 649.3134 1243.6861	610.3508	C_36_H_50_O_8_	610.3506	0.36	57672-63-6 [70,71,72,73]	Tigliane
**31**	17.78	[M+H]^+^ [M+Na]^+^ [M+K]^+^ [2M+Na]^+^	371.3156 393.2977 409.2714 763.6054	370.3085	C_22_H_42_O_4_	370.3083	0.38	/	/

* Components identified and confirmed using the molecular feature extraction (MFE) and find by formula algorithms of the MassHunter software (revision B.07.00), respectively. / Components that could not be tentatively identified by online literature search using the terms “*Euphorbia*, Euphorbiaceae” in SciFinder, an online database.

**Table 2:** Tentative identification of the components of the chloroform extract of the latex of *E. cyparissias* (EC) by LC-QToF MS according to the literature data available in SciFinder, an online database.

No.	RT (min)	Ion Species	*m*/*z* Measured	Molecular Mass Measured	Proposed Formula	Molecular Mass Calculated	Diff. (ppm)	Compound (CAS No.) [Ref.]	Class
**32**	1.31	[M+H]^+^	146.1177	145.1104	C_7_H_15_NO_2_	145.1103	0.77	407-64-7 [74] 1115-90-8 [75]	Amino acid Amino acid
**33**	3.33	[M+NH_4_]^+^ [M+Na]^+^ [M+K]^+^	648.3012 653.2569 669.2304	630.2676	C_33_H_42_O_12_	630.2676	−0.09	1811547-09-7 [76,77] 2049749-80-4 [78]	Jatrophane ent-Atisane
34 *	4.03	[M+NH_4_]^+^ [M+Na]^+^ [M+K]^+^	668.3059 673.2617 689.2357	650.2725	C_36_H_42_O_11_	650.2777	−0.32	1254956-17-6 [79,80,81] 1210299-33-4 [81] 2002494-82-6 [82]	Daphnane Daphnane Daphnane
35 *	4.15	[M+NH_4_]^+^ [M+Na]^+^ [M+K]^+^	588.2799 593.2356 609.2093	570.2463	C_31_H_38_O_10_	570.2465	−0.36	313486-57-6 [83] 313486-56-5 [83] 100288-19-5 [84] 2758418-28-7 [85] 2347529-35-3 [86] 1974283-21-0 [87]	Myrsinane Myrsinane Jatrophane Jatrophane Jatrophane Paraliane
36 *	5.27	[M+H]^+^ [M+Na]^+^ [M+K]^+^	676.2749 698.2570 714.2307	675.2676	C_37_H_41_NO_11_	675.2680	−0.50	2685765-74-4 [88] 2685765-73-3 [88] 2685762-55-2 [88]	Ingol Ingol Ingol
**37**	5.51	[M+NH_4_]^+^ [M+Na]^+^ [M+K]^+^ [2M+Na]^+^	630.2909 635.2462 651.2199 1247.5024	612.2570	C_33_H_40_O_11_	612.2571	−0.18	780755-68-2 [89,90] 709002-56-2 [90,91] 566189-66-0 [86,92] 2347529-24-0 [86,93] 2347529-23-9 [86] 220705-94-2 [94,95] 371974-77-5 [95] 313486-55-4 [83] 212842-87-0 [96] 557104-67-3 [97] 608525-82-2 [98] 616217-04-0 [99] 89984-07-6 [100] 2803346-38-3 [101]	Jatrophane Jatrophane Jatrophane Jatrophane Jatrophane Lathyrane Lathyrane Lathyrane Lathyrane Myrsinol Myrsinane Myrsinane Ingol Ingol
**38**	5.94	[M+NH_4_]^+^ [M+Na]^+^ [M+K]^+^ [2M+Na]^+^	710.3168 715.2726 731.2464 1407.5551	692.2832	C_38_H_44_O_12_	692.2833	−0.11	100198-29-6 [102] 100198-28-5 [102] 2051585-34-1 [77,103] 2051585-29-4 [103]	Jatrophane Jatrophane Jatrophane Jatrophane
**39**	5.97	[M+NH_4_]^+^ [M+Na]^+^ [M+K]^+^	754.3429 759.2986 775.2723	736.3093	C_40_H_48_O_13_	736.7395	−0.26	/	/
**40**	6.17	[M+NH_4_]^+^ [M+Na]^+^ [M+K]^+^ [2M+Na]^+^	710.3168 715.2722 731.2462 1407.5558	692.2830	C_38_H_44_O_12_	692.2833	−0.44	100198-29-6 [102] 100198-28-5 [102] 2051585-34-1 [77,103] 2051585-29-4 [103]	Jatrophane Jatrophane Jatrophane Jatrophane
**41**	6.42	[M+NH_4_]^+^ [M+Na]^+^ [M+K]^+^	690.3483 695.3040 711.2775	672.3147	C_36_H_48_O_12_	672.3146	0.14	/	/
**42**	6.95	[M+NH_4_]^+^ [M+Na]^+^ [M+K]^+^ [2M+Na]^+^	650.2952 655.2513 671.2250 1287.5141	632.2620	C_36_H_40_O_10_	632.2621	−0.20	2561483-25-6 [104]	Lathyrane
43 *	7.76	[M+H]^+^ [M+NH_4_]^+^ [M+Na]^+^ [M+K]^+^	720.3016 737.3355 742.2842 758.2579	719.2942	C_39_H_45_NO_12_	719.2942	0.37	2595240-63-2 [41] 2595235-60-0 [41] 2595235-05-3 [41] 2595233-36-4 [41] 777896-12-5 [42] 2408424-43-9 [43] 2342577-75-5 [44]	Jatrophane Jatrophane Jatrophane Jatrophane Jatrophane Premyrsinane Premyrsinane
44 *	7.88	[M+NH_4_]^+^ [M+Na]^+^ [M+K]^+^	692.3067 697.2621 713.2401	674.2734	C_38_H_42_O_11_	674.2727	0.97	2685775-35-1 [88,101] 2685775-67-9 [88] 2750352-31-7 [105] 2347529-31-9 [86]	Ingol Ingol Ingol Jatrophane
45 *	8.37	[M+NH_4_]^+^ [M+Na]^+^ [M+K]^+^	752.3276 757.2829 773.2562	734.2937	C_40_H_46_O_13_	734.2938	−0.23	2051585-33-0 [77,103] 2891708-35-1 [106]	Jatrophane Jatrophane
46 *	8.60	[M+NH_4_]^+^ [M+Na]^+^ [M+K]^+^	844.3174 849.2731 865.2466	826.2837	C_45_H_46_O_15_	826.2837	−0.01	2595253-64-6 [41]	Jatrophane
**47**	8.70	[M+H]^+^ [M+NH_4_]^+^ [M+Na]^+^ [M+K]^+^ [2M+Na]^+^ [2M+K]^+^	675.2774 692.3069 697.2625 713.2437 1371.5372 1387.5098	674.2747	C_38_H_42_O_11_	674.2727	2.97	2685775-35-1 [88,101] 2685775-67-9 [88] 2750352-31-7 [105] 2347529-31-9 [86]	Ingol Ingol Ingol Jatrophane
**48**	9.26	[M+H]^+^ [M+NH_4_]^+^ [M+Na]^+^ [M+K]^+^	717.2887 734.3177 739.2728 755.2465	716.2837	C_40_H_44_O_12_	716.2833	0.53	2685775-23-7 [88] 2685765-76-6 [88] 2347529-37-5 [86] 2347529-36-4 [86] 1342887-24-4 [93,107]	Ingol Ingol Jatrophane Jatrophane Jatrophane
49 *	9.36	[M+NH_4_]^+^ [M+Na]^+^ [M+K]^+^	812.3277 817.2833 833.2617	794.2944	C_45_H_46_O_13_	794.2938	0.71	/	/
**50**	9.47	[M+NH_4_]^+^ [M+Na]^+^ [M+K]^+^ [2M+Na]^+^	734.3173 739.2728 755.2576 1455.5590	716.2843	C_40_H_44_O_12_	716.2833	1.36	2685775-23-7 [88] 2685765-76-6 [88] 2347529-37-5 [86] 2347529-36-4 [86] 1342887-24-4 [93,107]	Ingol Ingol Jatrophane Jatrophane Jatrophane
**51**	9.66	[M+NH_4_]^+^ [M+Na]^+^ [M+K]^+^ [2M+Na]^+^	676.3117 681.2672 697.2416 1339.5422	658.2779	C_38_H_42_O_10_	658.2778	0.19	2366129-51-1 [60] 2366129-44-2 [60] 2750352-32-8 [105] 1613699-93-6 [108] 1151831-79-6 [109]	Myrsinane Myrsinane Ingol Ingol Ingol
**52**	9.70	[M+NH_4_]^+^ [M+Na]^+^ [M+K]^+^	856.3539 861.3090 877.2825	838.3198	C_47_H_50_O_14_	838.3201	−0.29	/	/
**53**	10.02	[M+Na]^+^ [M+K]^+^ [2M+Na]^+^	579.2356 595.2091 1135.4805	556.2463	C_34_H_36_O_7_	556.2461	0.32	59086-90-7 [110] 91413-70-6 [111] 91413-69-3 [111] 174389-91-4 [112] 92118-01-9 [113]	Ingenane Ingenane Ingenane Ingenane Tigliane
**54**	10.19	[M+NH_4_]^+^ [M+Na]^+^ [M+K]^+^	714.3484 719.3038 735.2776	696.3145	C_38_H_48_O_12_	696.3146	−0.07	284666-41-7 [114] 606136-90-7 [115] 1977558-48-7 [57]	Jatrophane Jatrophane Myrsinane
55 *	10.30	[M+NH_4_]^+^ [M+Na]^+^ [M+K]^+^	772.3330 777.2879 793.2610	754.2985	C_43_H_46_O_12_	754.2989	−0.03	1449465-16-0 [80]	Daphnane
**56**	10.33	[M+NH_4_]^+^ [M+Na]^+^ [M+K]^+^	714.3486 719.3039 735.2774	696.3147	C_38_H_48_O_12_	696.3146	0.14	284666-41-7 [114] 606136-90-7 [115] 1977558-48-7 [57]	Jatrophane Jatrophane Myrsinane
**57**	10.43	[M+NH_4_]^+^ [M+Na]^+^ [M+K]^+^	760.3326 765.2876 781.2606	742.2985	C_42_H_46_O_12_	742.2989	−0.52	/	/
**58**	10.47	[M+NH_4_]^+^ [M+Na]^+^ [M+K]^+^	600.3164 605.2721 621.2459	582.2828	C_33_H_42_O_9_	582.2829	−0.13	81557-52-0 [116] 126372-45-0 [117] 126372-52-9 [117] 126372-50-7 [117] 515854-87-2 [118] 515854-85-0 [118] 515854-83-8 [118] 1253641-57-4 [119] 586971-22-4 [90,120] 1010414-43-3 [121] 944799-48-8 [122]	Jatrophane Jatrophane Jatrophane Jatrophane Jatrophane Jatrophane Jatrophane Jatrophane Jatrophane Jatrophane Lathyrane
**59**	10.49	[M+NH_4_]^+^ [M+Na]^+^	656.3428 661.2979	638.3087	C_36_H_46_O_10_	638.3091	−0.66	/	/
60 *	10.54	[M+NH_4_]^+^ [M+Na]^+^ [M+K]^+^	720.3378 725.2933 741.2679	702.3040	C_40_H_46_O_11_	702.3040	−0.06	/	/
**61**	11.33	[M+NH_4_]^+^ [M+Na]^+^ [M+K]^+^	796.3330 801.2882 817.2620	778.2991	C_45_H_46_O_12_	778.2989	0.22	/	/
**62**	11.41	[M+H]^+^ [M+Na]^+^ [M+K]^+^ [2M+Na]^+^	551.2998 573.2823 589.2584 1123.5752	550.2931	C_33_H_42_O_7_	550.2931	0.11	1010806-00-4 [122] 1811530-78-5 [123] 62820-23-9 [124]	Ingenane Ingenane Lathyrane
63 *	11.64	[M+Na]^+^ [M+K]^+^	527.3705 543.3547	504.3819	C_31_H_52_O_5_	504.3815	0.85	/	/
64 *	12.51	[M+NH_4_]^+^ [M+Na]^+^	812.3276 817.2827	794.2936	C_45_H_46_O_13_	794.2938	−0.27	/	/
**65**	12.81	[M+H]^+^ [M+NH_4_]^+^ [M+Na]^+^ [M+K]^+^ [2M+NH_4_]^+^ [2M+Na]^+^	545.3471 562.3685 567.3292 583.3033 1106.7130 1111.6693	544.3400	C_32_H_48_O_7_	544.3400	-0.08	478243-87-7 [125] 92117-95-8 [113] 1020102-66-2 [126] 100217-91-2 [127]	Ingenane Tigliane Tigliane Tigliane
**66**	12.96	[M+H]^+^ [M+NH_4_]^+^ [M+Na]^+^	675.4103 692.4372 697.3922	674.4034	C_38_H_58_O_10_	674.4030	0.61	76663-59-7 [30] 76663-58-6 [30] 76663-57-5 [30] 1362115-49-8 [128]	Ingenane Ingenane Ingenane Tigliane
**67**	13.66	[M+H]^+^ [M+NH_4_]^+^ [M+Na]^+^ [M+K]^+^ [2M+NH_4_]^+^ [2M+Na]^+^	623.3578 640.3875 645.3431 661.3140 1262.7365 1267.6896	622.3507	C_37_H_50_O_8_	622.3506	0.20	149725-35-9 [129]	Daphnane
68 *	14.03	[M+Na]^+^ [M+K]^+^	455.3518 471.3101	454.3453	C_30_H_46_O_3_	454.3447	1.31	125456-55-5 [130,131] 125456-62-4 [130] 132831-05-1 [131] 94530-05-9 [132] 242814-44-4 [133] 1000000-03-2 [134] 1000000-04-3 [134] 2411214-36-1 [135] 2727156-37-6 [136] 2101307-34-8 [137]	Triterpene Triterpene Triterpene Triterpene Triterpene Triterpene Triterpene Triterpene Triterpene Triterpene
69 *	14.22	[M+H]^+^ [M+NH_4_]^+^ [M+Na]^+^ [M+K]^+^	651.4051 668.4155 673.3711 689.3442	650.3821	C_39_H_54_O_8_	650.3819	0.30	184221-48-5 [138] 184221-44-1 [138]	Ingenane Ingenane
70 *	14.59	[M+H]^+^ [M+NH_4_]^+^ [M+Na]^+^ [M+K]^+^	667.4206 684.4463 689.4023 705.3752	666.4132	C_40_H_58_O_8_	666.4132	−0.02	/	/
**71**	14.98	[M+H]^+^ [M+Na]^+^ [M+K]^+^	617.4034 639.3868 655.3600	616.3975	C_36_H_56_O_8_	616.3975	−0.11	76663-56-4 [30] 1333380-60-1 [139]	Ingenane Ingenane
**72**	15.63	[M+H]^+^ [M+NH_4_]^+^ [M+Na]^+^ [M+K]^+^ [2M+NH_4_]^+^ [2M+Na]^+^	651.3885 668.4215 673.3714 689.3450 1318.8020 1323.7525	650.3826	C_39_H_54_O_8_	650.3819	1.14	184221-48-5 [138] 184221-44-1 [138]	Ingenane Ingenane
73 *	15.84	[M+H]^+^ [M+Na]^+^ [M+K]^+^	631.4203 653.4026 669.3765	630.4133	C_37_H_58_O_8_	630.4132	0.23	57716-89-9 [140] 182997-47-3 [141]	Ingenane Tigliane
74 *	16.18	[M+H]^+^	441.3727	440.3655	C_30_H_48_O_2_	440.3654	0.06	142449-67-0 [131] 242814-43-3 [133] 242814-43-3 [133] 2067-65-4 [142] 110011-56-8 [34,142] 112406-53-8 [142] 122272-22-4 [143] 1650569-06-4 [144] 2413472-28-1 [145] 38242-02-3 [146] 6060-07-7 [146] 2852676-92-5 [146] 3866-77-1 [146,147] 543691-16-3 [148] 543691-17-4 [149] 543691-19-6 [149] 22478-71-3 [150] 1384465-02-4 [151] 138994-69-1 [152] 2004651-44-7 [153] 13159-28-9 [154]	Triterpene Triterpene Triterpene Triterpene Triterpene Triterpene Triterpene Triterpene Triterpene Triterpene Triterpene Triterpene Triterpene Triterpene Triterpene Triterpene Triterpene Triterpene Triterpene Triterpene Triterpene
**75**	17.25	[M+H]^+^ [M+NH_4_]^+^ [M+Na]^+^ [M+K]^+^	653.5347 670.5604 675.5170 691.4930	652.5276	C_39_H_72_O_7_	652.5278	−0.34	/	/
**76**	17.28	[M+Na]^+^ [M+K]^+^	623.3920 639.3661	600.4028	C_36_H_56_O_7_	600.4026	0.33	1020102-70-8 [126] 349152-28-9 [155]	Tigliane Tigliane
**77**	17.59	[M+H]^+^ [M+Na]^+^ [M+K]^+^ [2M+NH_4_]^+^ [2M+Na]^+^	645.4368 667.4182 683.3931 1306.8935 1311.8487	644.4290	C_38_H_60_O_8_	644.4288	0.27	76663-53-1 [30] 76663-55-3 [30] 76663-54-2 [30] 54706-69-3 [139,156] 2254317-50-3 [157] 20839-12-7 [128,158] 67492-54-0 [158] 73089-77-7 [158]	Ingenane Ingenane Ingenane Ingenane Ingenane Tigliane Tigliane Tigliane
78 *	17.78	[M+H]^+^ [M+NH_4_]^+^ [M+Na]^+^ [M+K]^+^	371.3155 388.3419 393.2975 409.2714	370.3083	C_22_H_42_O_4_	370.3083	−0.08	/	/
79 *	18.25	[M+NH_4_]^+^ [M+Na]^+^ [M+K]^+^	652.4261 657.3764 673.3503	634.3871	C_39_H_54_O_7_	634.3870	0.26	/	*/*
**80**	21.18	[M+H]^+^ [M+Na]^+^ [M+K]^+^	629.4391 651.4233 667.3972	628.4340	C_38_H_60_O_7_	628.4339	0.21	672945-80-1 [128,138] 1407160-19-3 [159] 1020102-72-0 [126]	Ingenane Ingenane Tigliane

* Components identified using the molecular feature extraction (MFE) and find by formula algorithms of the MassHunter software (revision B.07.00), respectively. / Components that could not be tentatively identified by online literature search using the terms “*Euphorbia*, Euphorbiaceae” in SciFinder, an online database.

**Table 3:** Cell growth inhibition induced by the EC and ES extracts.

Extract	IC_50_, μg mL^−1^				
	NCI-H460	NCI-H460/R	U87	U87-TxR	MRC-5
EC	8.89 ± 2.55	33.48 ± 8.90	12.96 ± 4.14	12.22 ± 4.23	6.55 ± 2.64
ES	20.11 ± 6.38	37.99 ± 18.72	15.71 ± 4.57	17.26 ± 4.10	5.89 ± 2.21

## Data Availability

The data is contained within the manuscript.

## Supplementary Materials

The following supporting information can be downloaded at https://www.mdpi.com/article/10.3390/plants12244181/s1. Figures S1–S31: ESI (+) mass spectra of components **1**–**31**, with the corresponding retention times (Table 1), obtained from the chloroform extract of the latex of *E. seguieriana* ssp. *seguieriana* Necker (ES); Figures S32–S79: ESI (+) mass spectra of components **33**–**80**, with the corresponding retention times (Table 2), obtained from the chloroform extract of the latex of *E. cyparissias* (EC).
