# Supplementary material for: LC-ESI QToF MS Non-Targeted Screening of Latex Extracts of Euphorbia seguieriana ssp. seguieriana Necker and Euphorbia cyparissias and Determination of Their Potential Anticancer Activity
Source: Plants (Basel). 2023 Dec 16;12(24):4181. doi: 10.3390/plants12244181 (PMC10747863; doi:10.3390/plants12244181)
Supplement: Supplementary file 1 [file plants-12-04181-s001.zip › plants-2712188-supplementary.pdf]

plants MDPI

Supplementary Materials

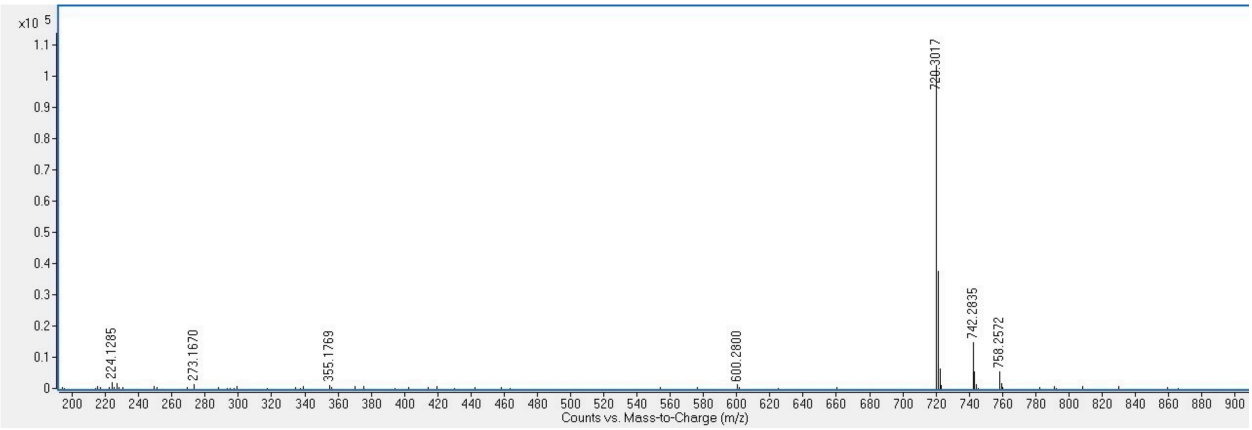

Figure S1. ESI (+) mass spectrum of component **1** eluting at t_R = 5.26 min in the chloroform extract of the latex of *E. seguieriana* ssp. *seguieriana* Necker (ES).

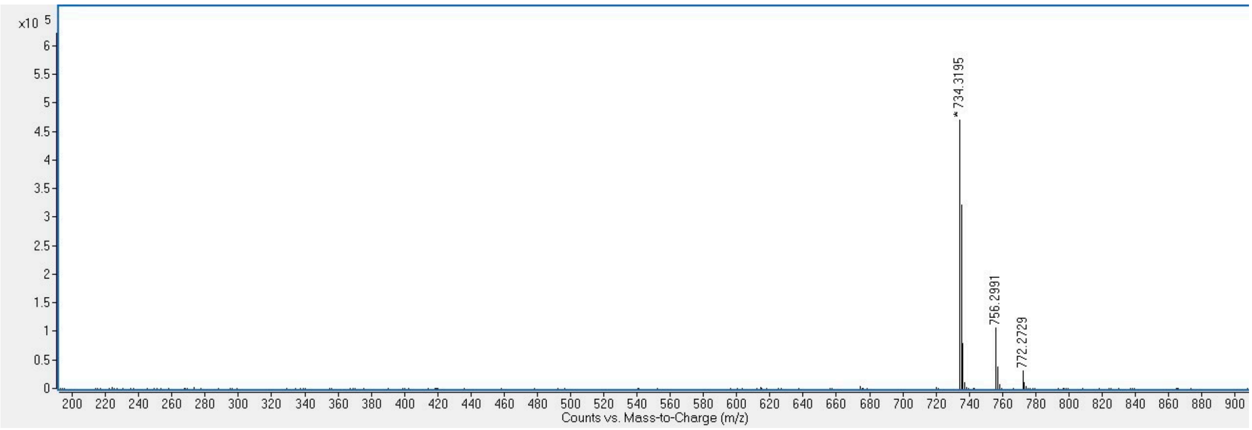

Figure S2. ESI (+) mass spectrum of component **2** eluting at t_R = 6.27 min in the chloroform extract of the latex of *E. seguieriana* ssp. *seguieriana* Necker (ES).

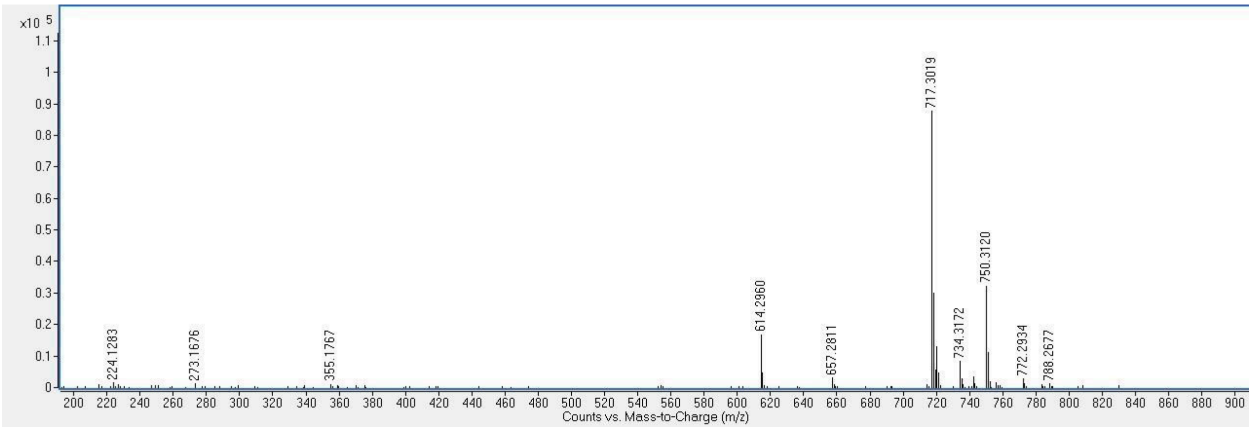

Figure S3. ESI (+) mass spectrum of component **3** eluting at t_R = 6.49 min in the chloroform extract of the latex of *E. seguieriana* ssp. *seguieriana* Necker (ES).

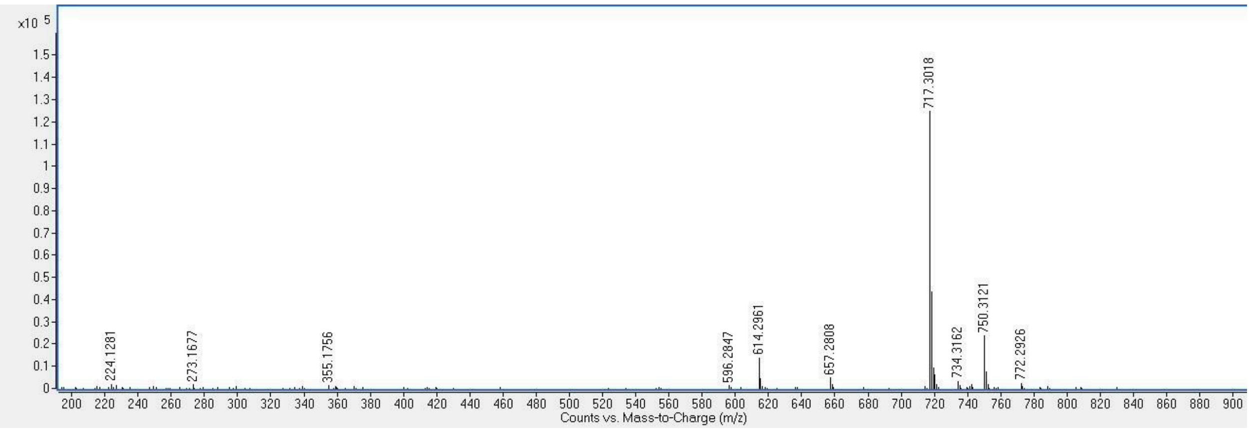

Figure S4. ESI (+) mass spectrum of component **4** eluting at t_R = 6.52 min in the chloroform extract of the latex of *E. seguieriana* ssp. *seguieriana* Necker (ES).

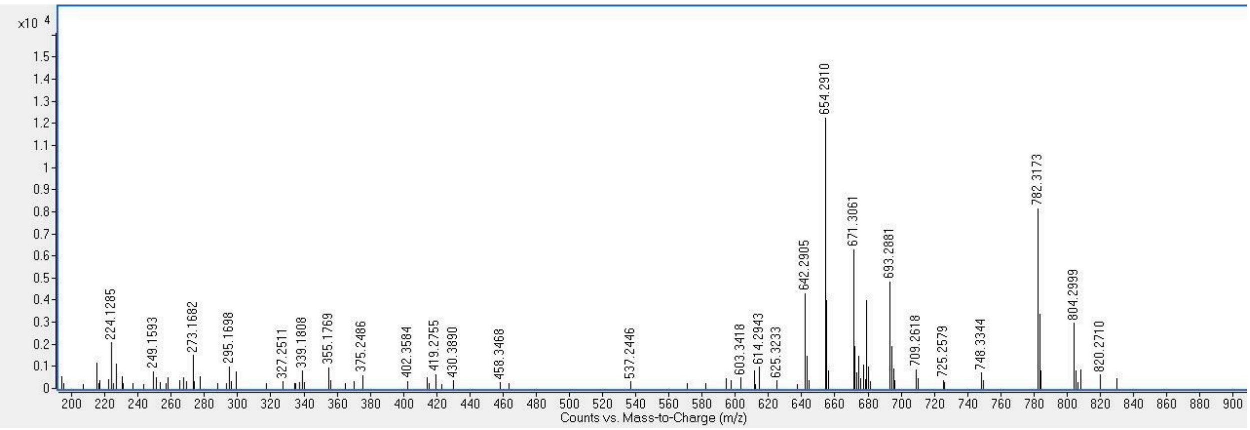

Figure S5. ESI (+) mass spectrum of component **5** eluting at t_R = 7.07 min in the chloroform extract of the latex of *E. seguieriana* ssp. *seguieriana* Necker (ES).

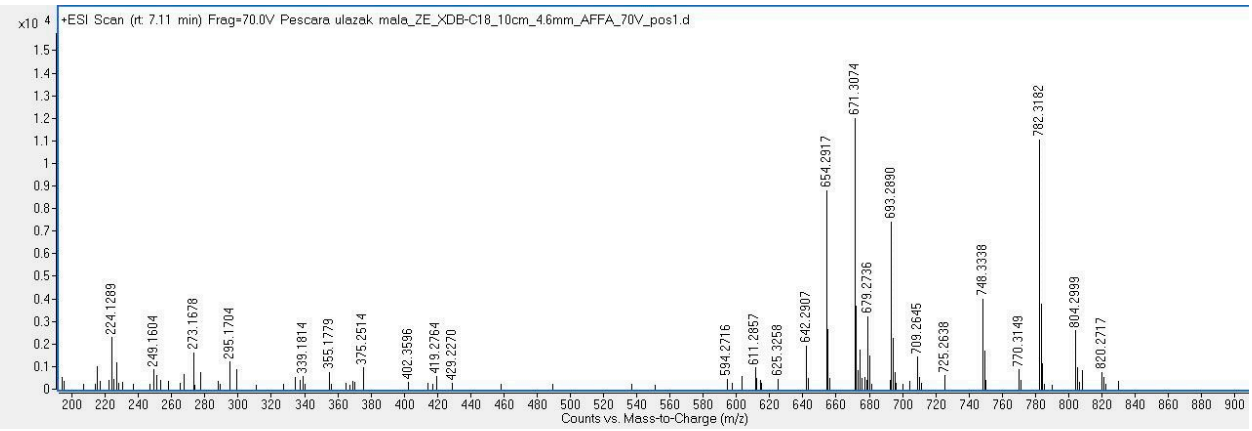

Figure S6. ESI (+) mass spectrum of component **6** eluting at t_R = 7.11 min in the chloroform extract of the latex of *E. seguieriana* ssp. *seguieriana* Necker (ES).

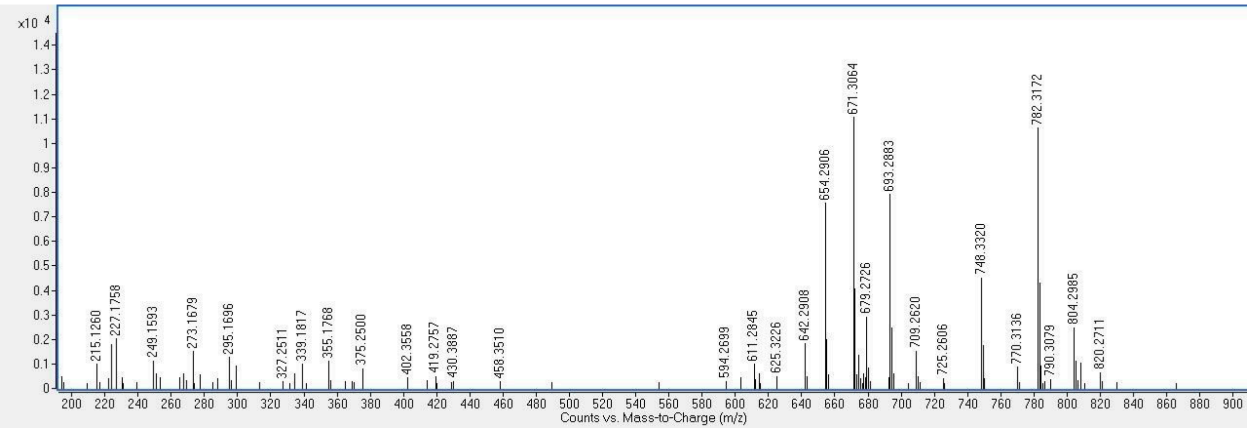

Figure S7. ESI (+) mass spectrum of component **7** eluting at t_R = 7.12 min in the chloroform extract of the latex of *E. seguieriana* ssp. *seguieriana* Necker (ES).

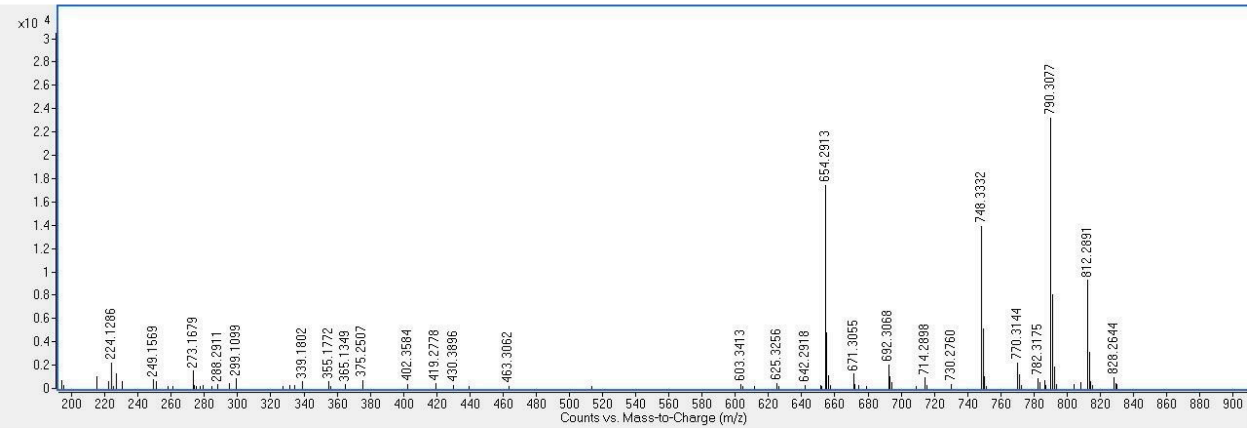

Figure S8. ESI (+) mass spectrum of component **8** eluting at t_R = 7.24 min in the chloroform extract of the latex of *E. seguieriana* ssp. *seguieriana* Necker (ES).

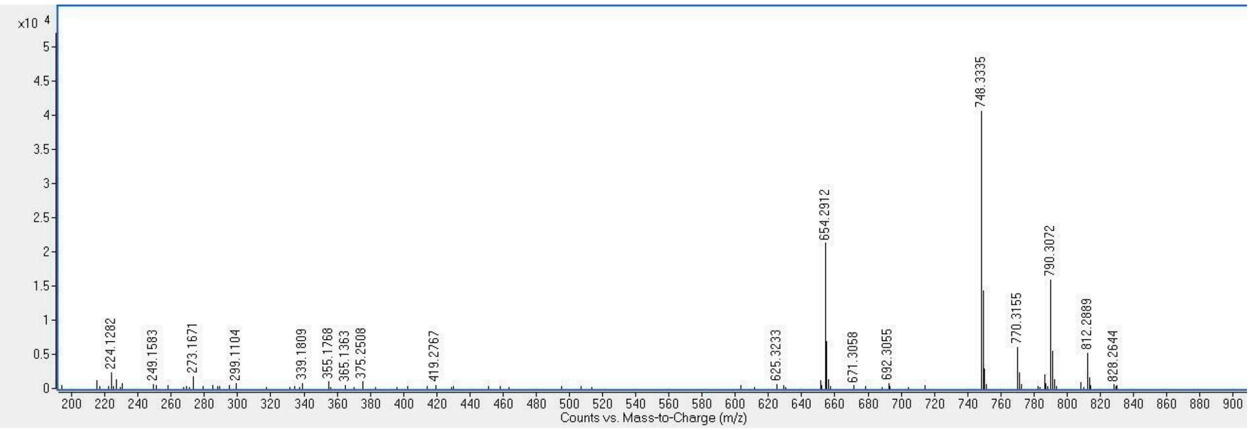

Figure S9. ESI (+) mass spectrum of component **9** eluting at t_R = 7.28 min in the chloroform extract of the latex of *E. seguieriana* ssp. *seguieriana* Necker (ES).

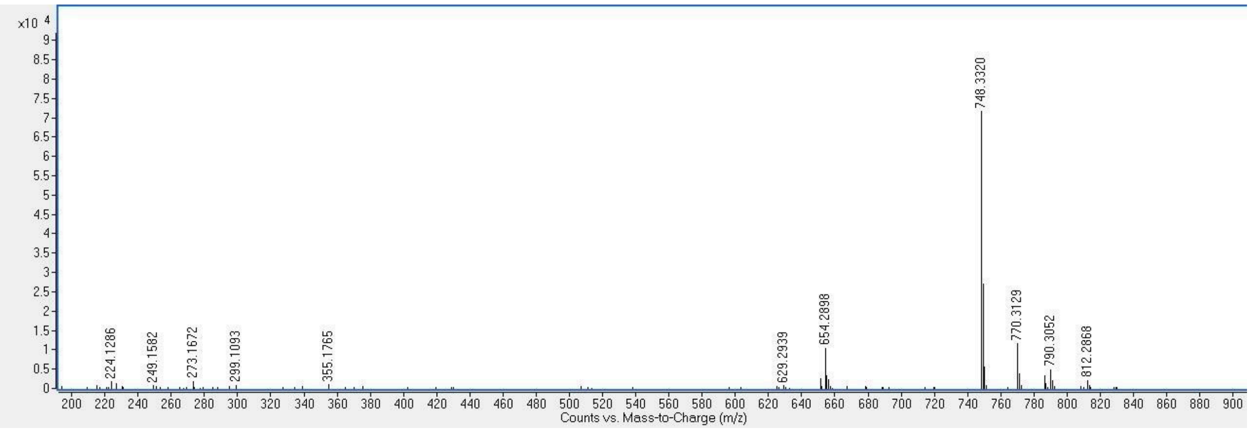

Figure S10. ESI (+) mass spectrum of component **10** eluting at t_R = 7.33 min in the chloroform extract of the latex of *E. seguieriana* ssp. *seguieriana* Necker (ES).

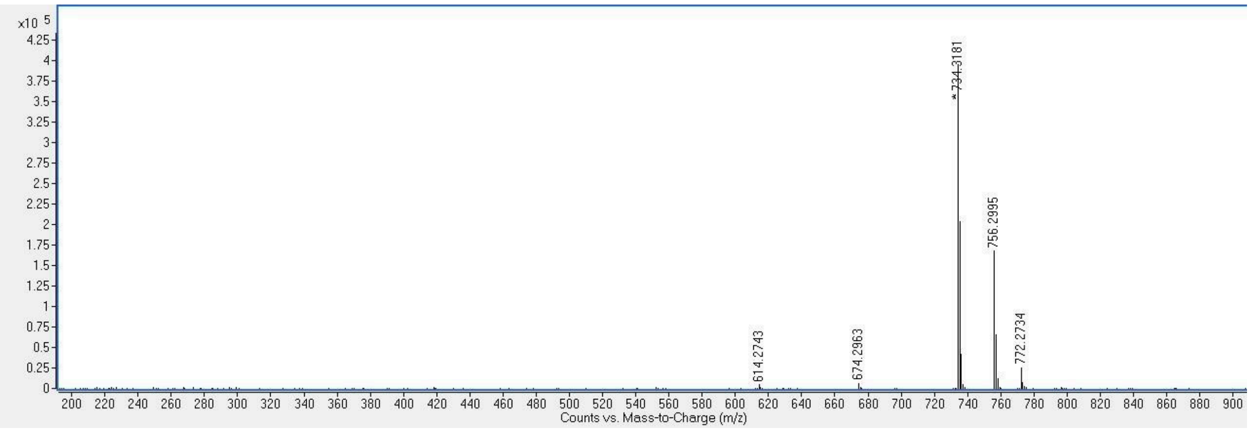

Figure S11. ESI (+) mass spectrum of component **11** eluting at t_R = 7.67 min in the chloroform extract of the latex of *E. seguieriana* ssp. *seguieriana* Necker (ES).

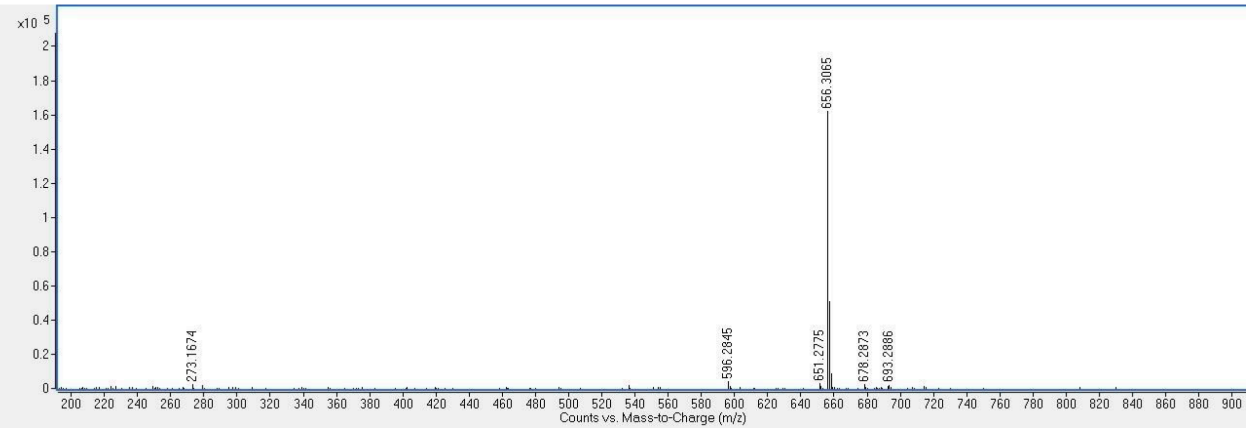

Figure S12. ESI (+) mass spectrum of component **12** eluting at t_R = 8.12 min in the chloroform extract of the latex of *E. seguieriana* ssp. *seguieriana* Necker (ES).

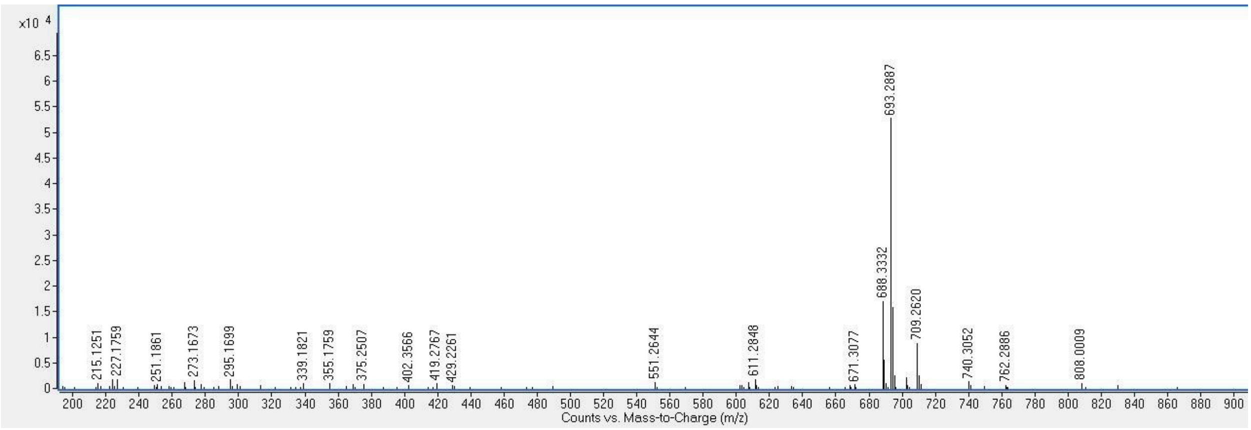

Figure S13. ESI (+) mass spectrum of component **13** eluting at t_R = 8.39 min in the chloroform extract of the latex of *E. seguieriana* ssp. *seguieriana* Necker (ES).

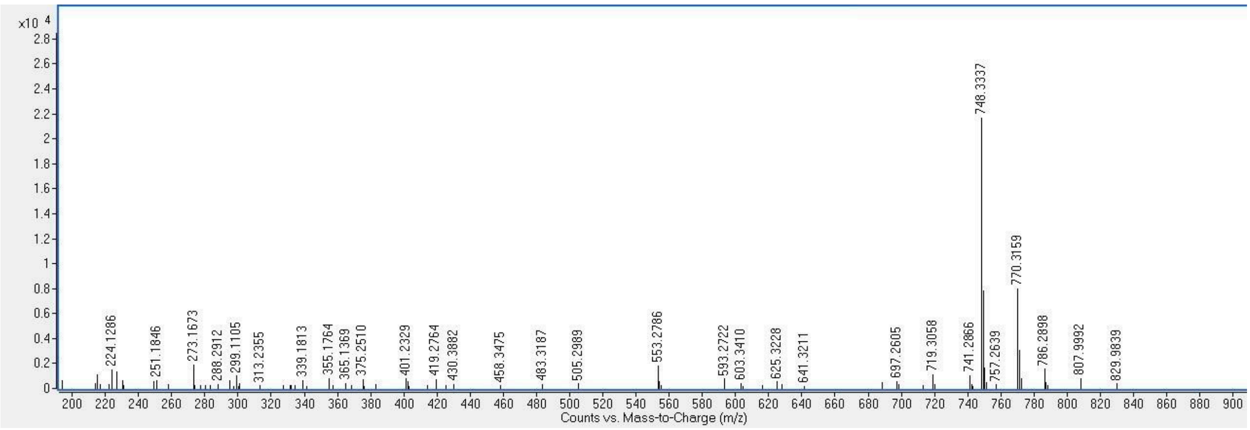

Figure S14. ESI (+) mass spectrum of component **14** eluting at t_R = 8.77 min in the chloroform extract of the latex of *E. seguieriana* ssp. *seguieriana* Necker (ES).

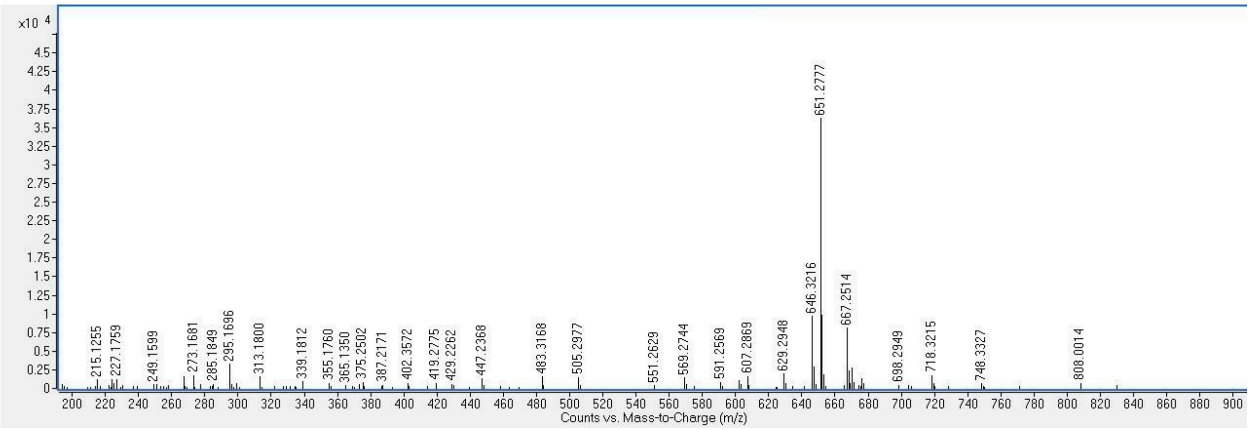

Figure S15. ESI (+) mass spectrum of component **15** eluting at t_R = 9.08 min in the chloroform extract of the latex of *E. seguieriana* ssp. *seguieriana* Necker (ES).

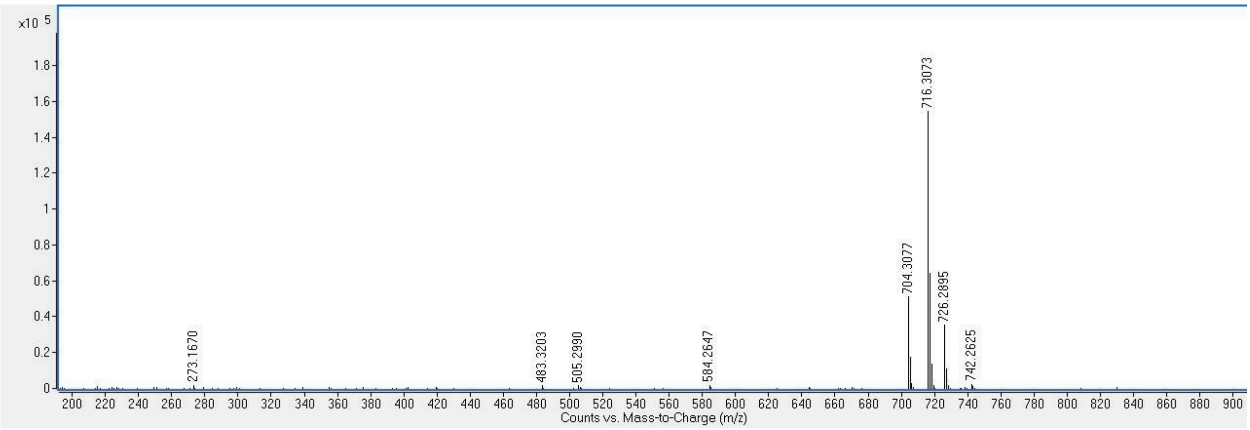

Figure S16. ESI (+) mass spectrum of component **16** eluting at t_R = 9.34 min in the chloroform extract of the latex of *E. seguieriana* ssp. *seguieriana* Necker (ES).

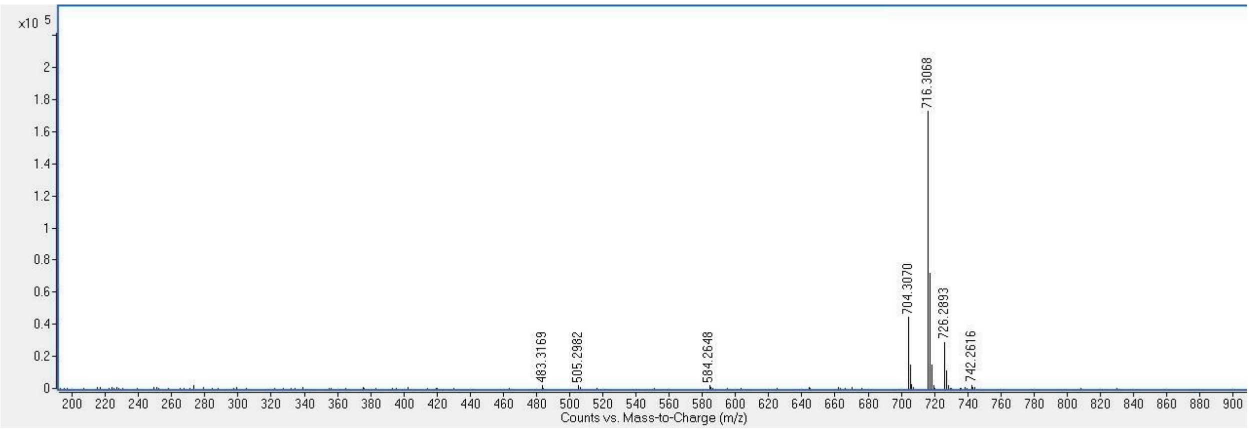

Figure S17. ESI (+) mass spectrum of component **17** eluting at t_R = 9.36 min in the chloroform extract of the latex of *E. seguieriana* ssp. *seguieriana* Necker (ES).

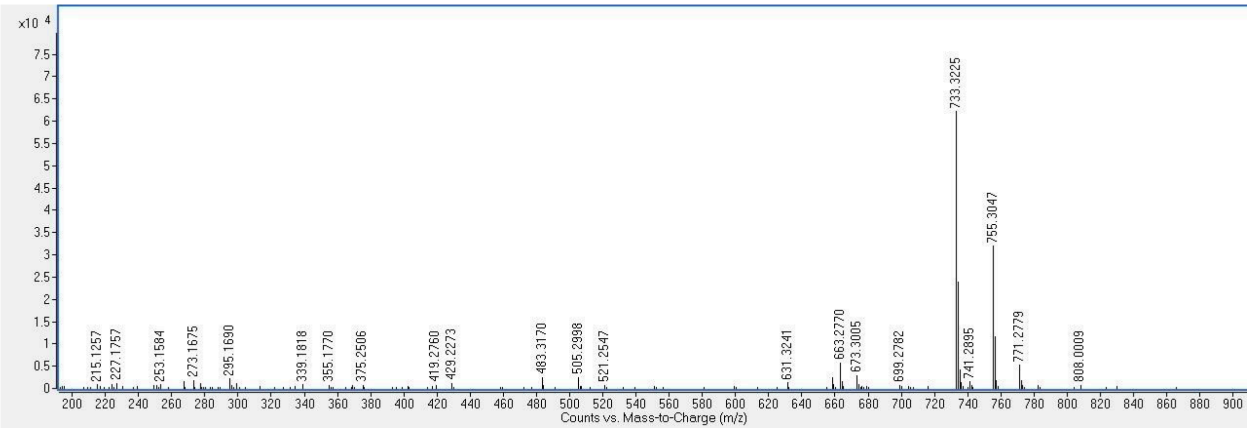

Figure S18. ESI (+) mass spectrum of component **18** eluting at t_R = 9.62 min in the chloroform extract of the latex of *E. seguieriana* ssp. *seguieriana* Necker (ES).

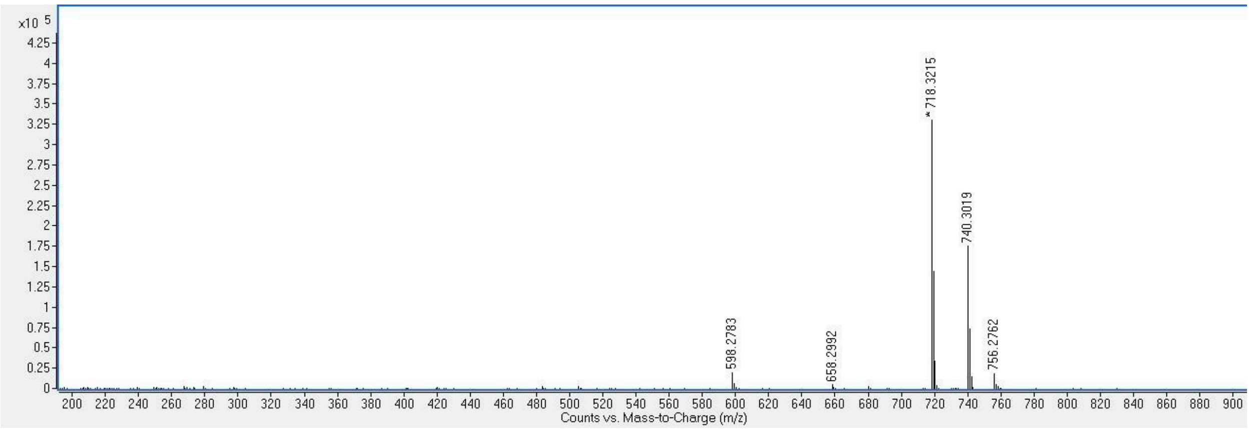

Figure S19. ESI (+) mass spectrum of component **19** eluting at t_R = 10.21 min in the chloroform extract of the latex of *E. seguieriana* ssp. *seguieriana* Necker (ES).

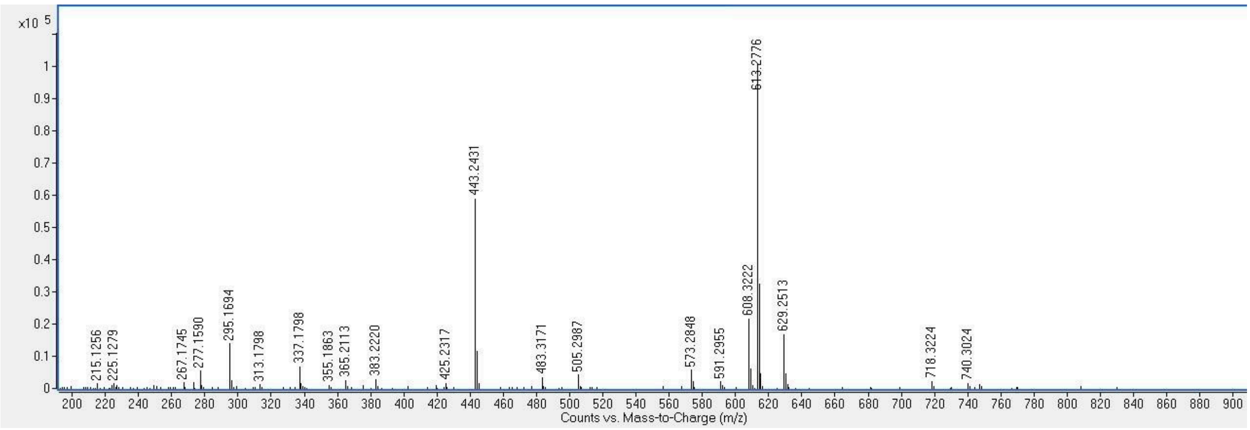

Figure S20. ESI (+) mass spectrum of component **20** eluting at t_R = 10.43 min in the chloroform extract of the latex of *E. seguieriana* ssp. *seguieriana* Necker (ES).

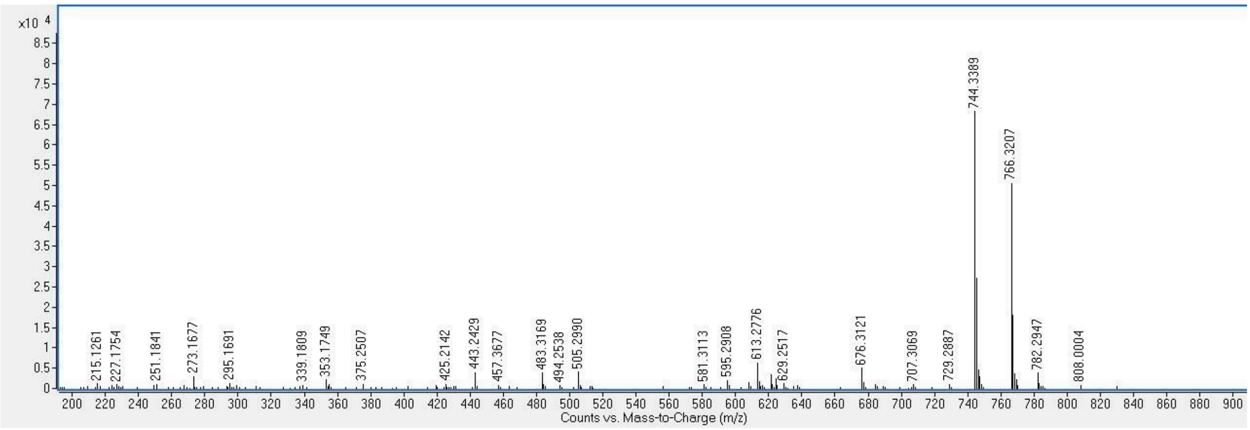

Figure S21. ESI (+) mass spectrum of component **21** eluting at t_R = 10.57 min in the chloroform extract of the latex of *E. seguieriana* ssp. *seguieriana* Necker (ES).

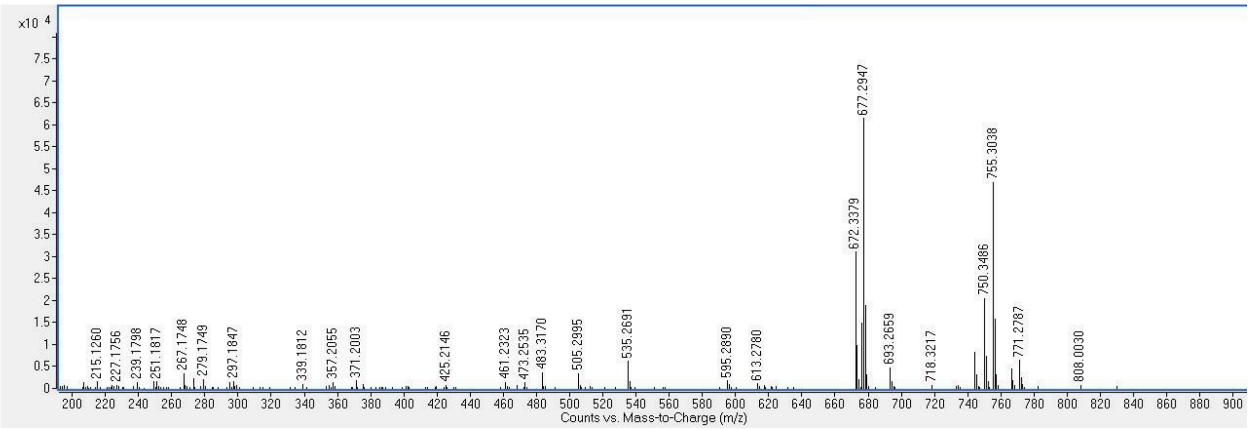

Figure S22. ESI (+) mass spectrum of component **22** eluting at t$_R$ = 10.69 min in the chloroform extract of the latex of *E. seguieriana* ssp. *seguieriana* Necker (ES).

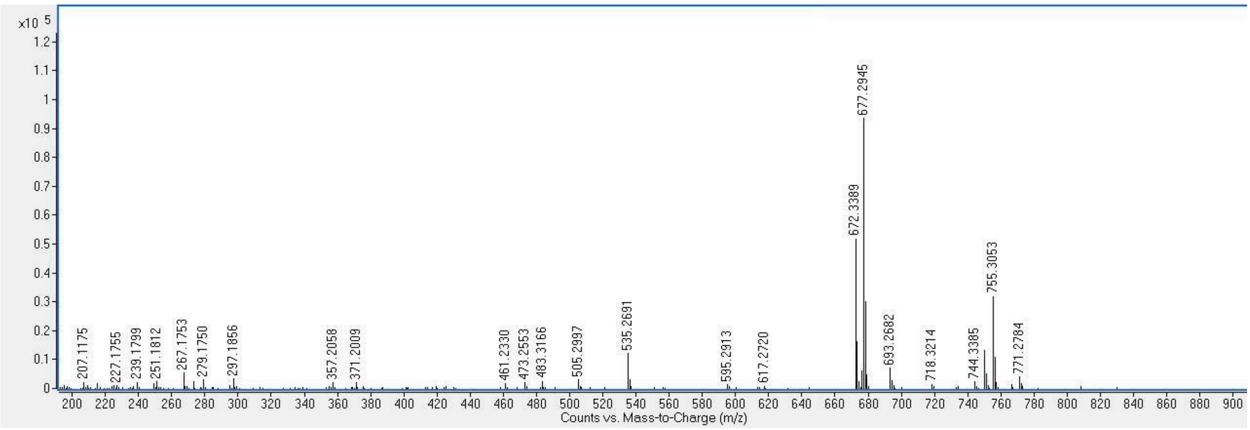

Figure S23. ESI (+) mass spectrum of component **23** eluting at t$_R$ = 10.73 min in the chloroform extract of the latex of *E. seguieriana* ssp. *seguieriana* Necker (ES).

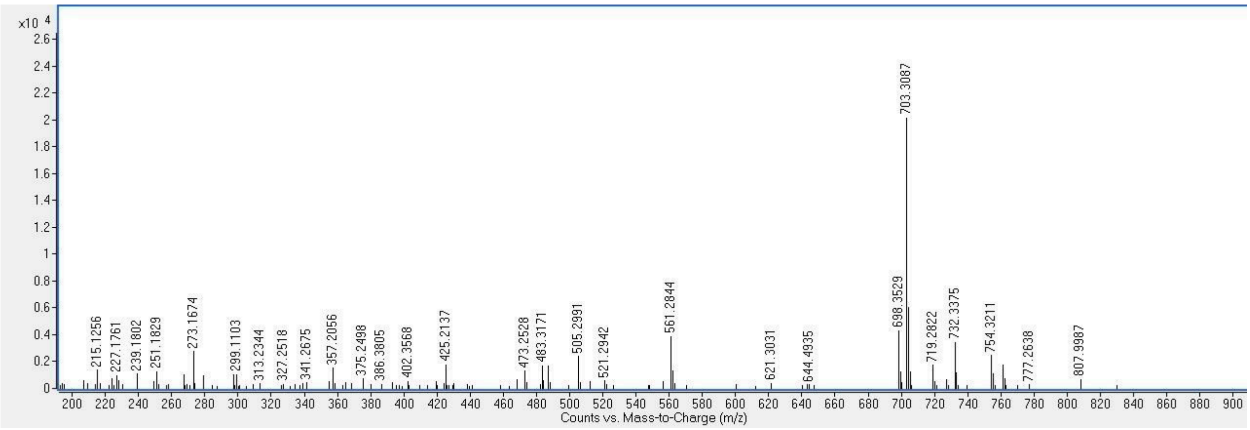

Figure S24. ESI (+) mass spectrum of component **24** eluting at t$_R$ = 11.06 min in the chloroform extract of the latex of *E. seguieriana* ssp. *seguieriana* Necker (ES).

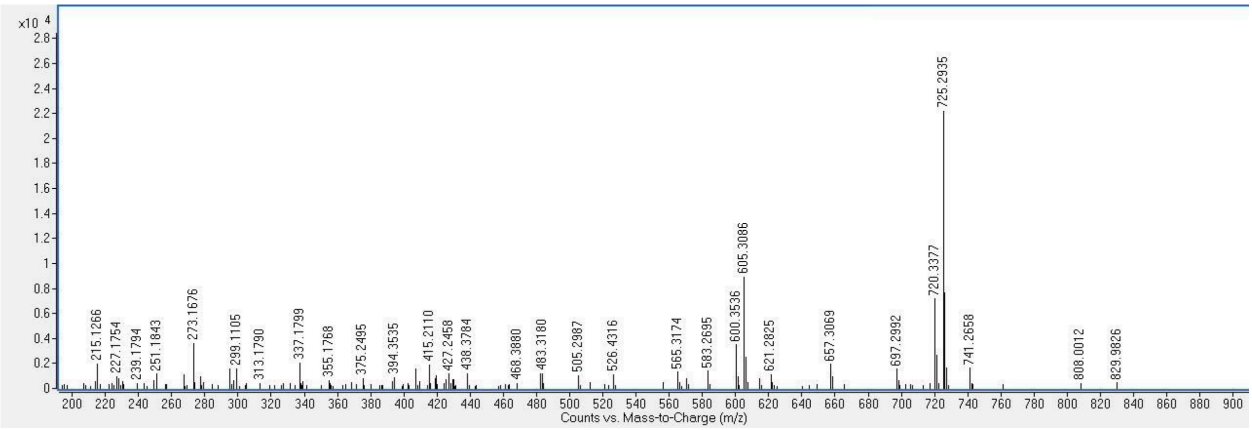

Figure S25. ESI (+) mass spectrum of component **25** eluting at t_R = 11.95 min in the chloroform extract of the latex of *E. seguieriana* ssp. *seguieriana* Necker (ES).

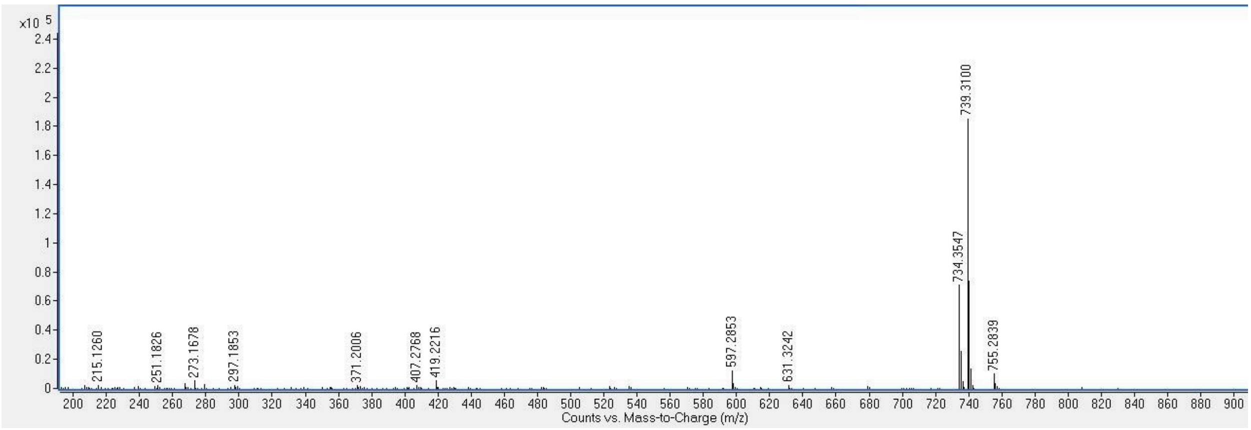

Figure S26. ESI (+) mass spectrum of component **26** eluting at t_R = 12.70 min in the chloroform extract of the latex of *E. seguieriana* ssp. *seguieriana* Necker (ES).

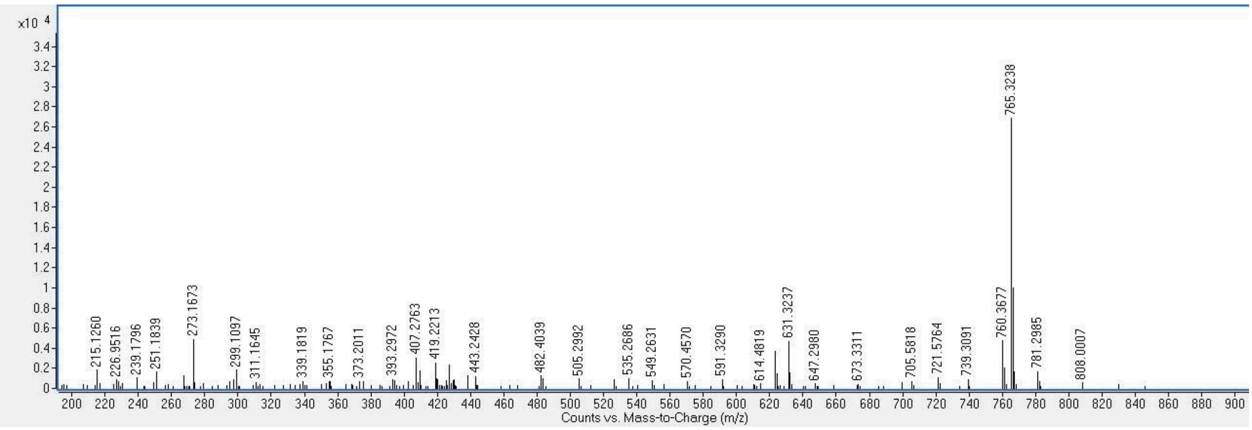

Figure S27. ESI (+) mass spectrum of component **27** eluting at t_R = 12.89 min in the chloroform extract of the latex of *E. seguieriana* ssp. *seguieriana* Necker (ES).

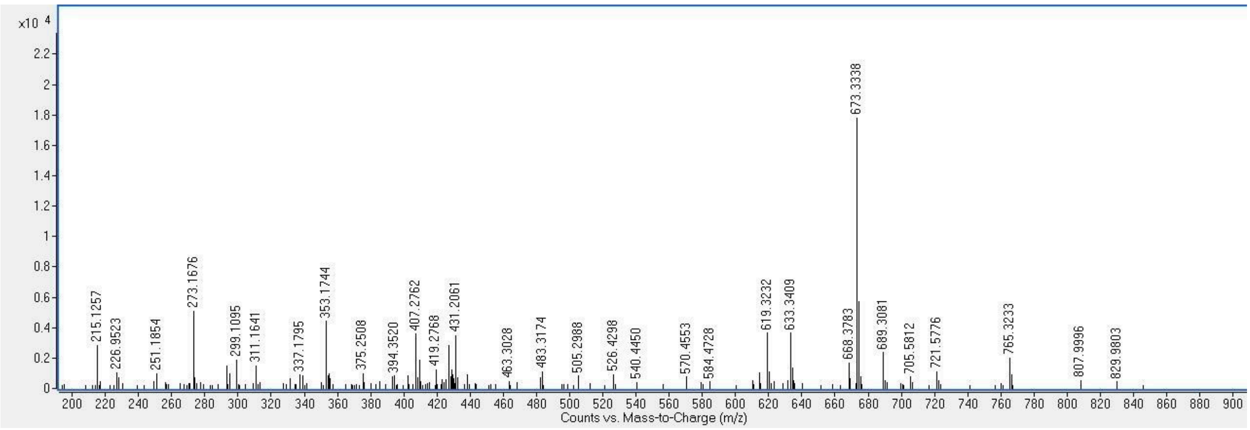

Figure S28. ESI (+) mass spectrum of component **28** eluting at t*R* = 13.01 min in the chloroform extract of the latex of *E. seguieriana* ssp. *seguieriana* Necker (ES).

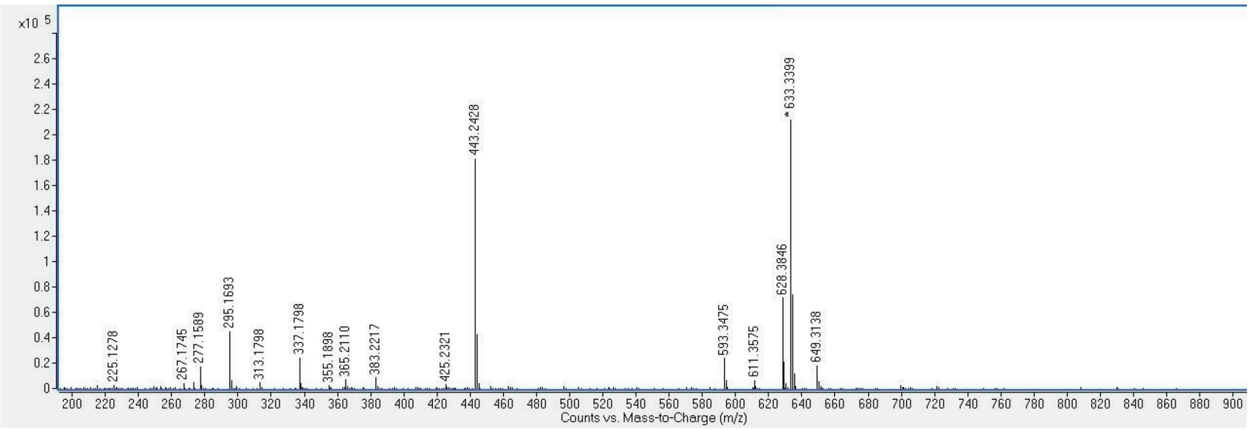

Figure S29. ESI (+) mass spectrum of component **29** eluting at t*R* = 13.92 min in the chloroform extract of the latex of *E. seguieriana* ssp. *seguieriana* Necker (ES).

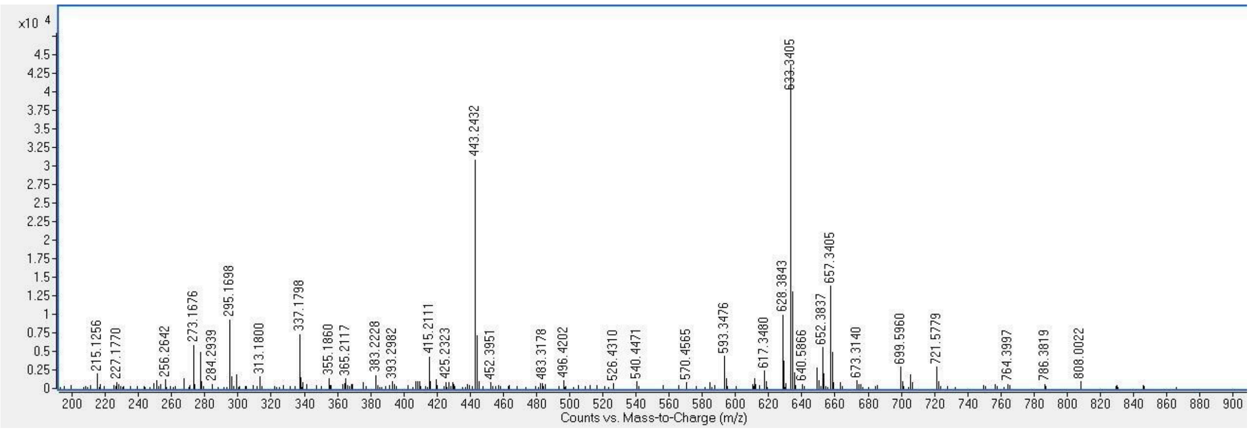

Figure S30. ESI (+) mass spectrum of component **30** eluting at t*R* = 14.13 min in the chloroform extract of the latex of *E. seguieriana* ssp. *seguieriana* Necker (ES).

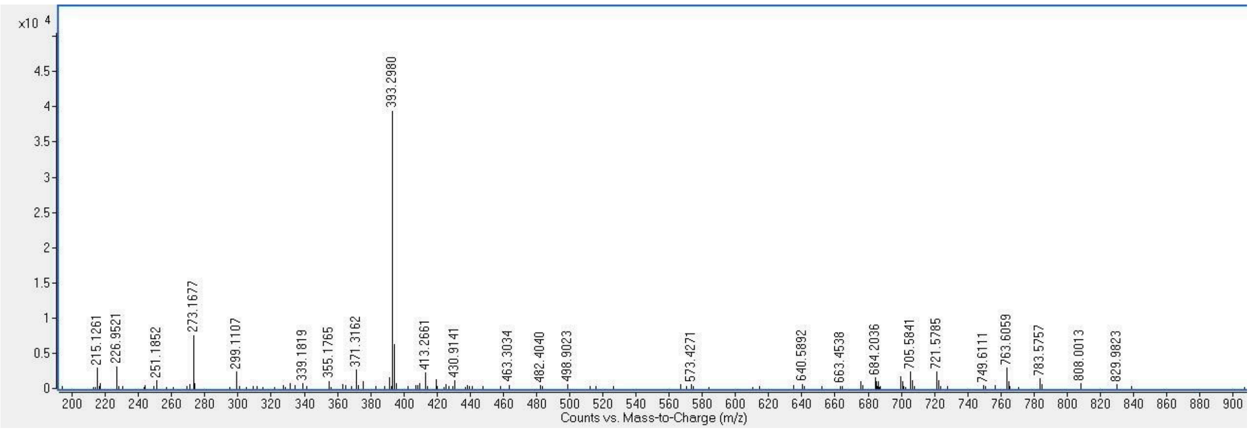

Figure S31. ESI (+) mass spectrum of component **31** eluting at t_R = 17.78 min in the chloroform extract of the latex of *E. seguieriana* ssp. *seguieriana* Necker (ES).

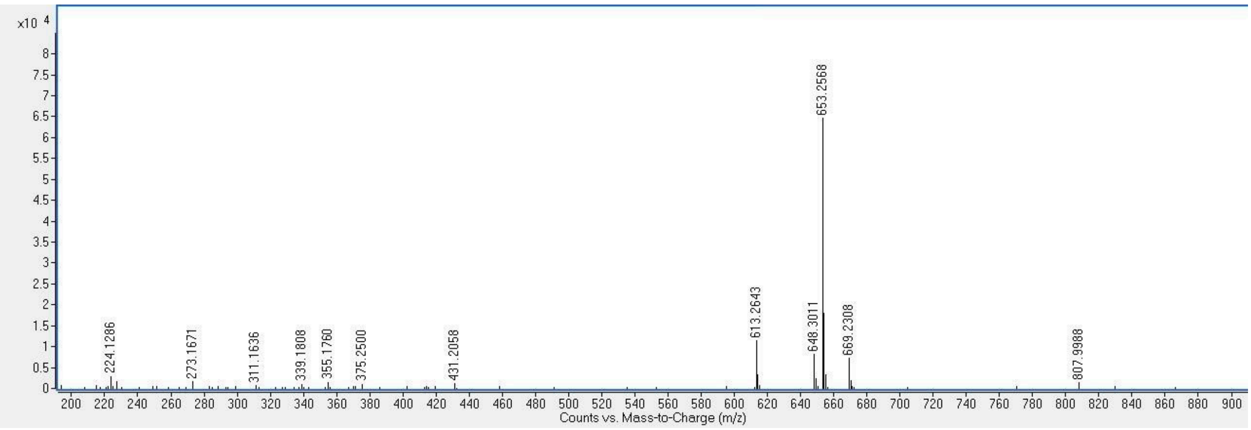

Figure S32. ESI (+) mass spectrum of component **33** eluting at t_R = 3.33 min in the chloroform extract of the latex of *E. cyparissias* (EC).

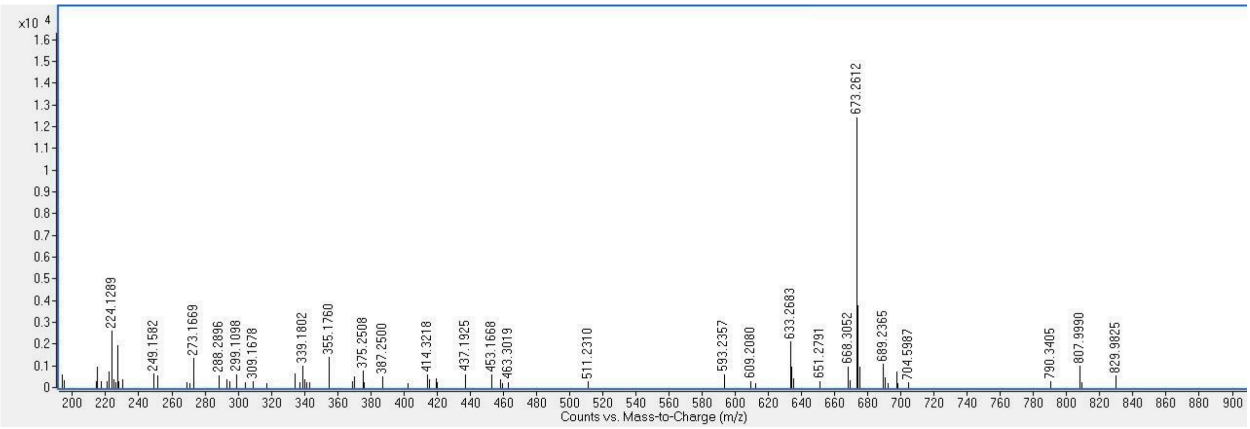

Figure S33. ESI (+) mass spectrum of component **34** eluting at t_R = 4.03 min in the chloroform extract of the latex of *E. cyparissias* (EC).

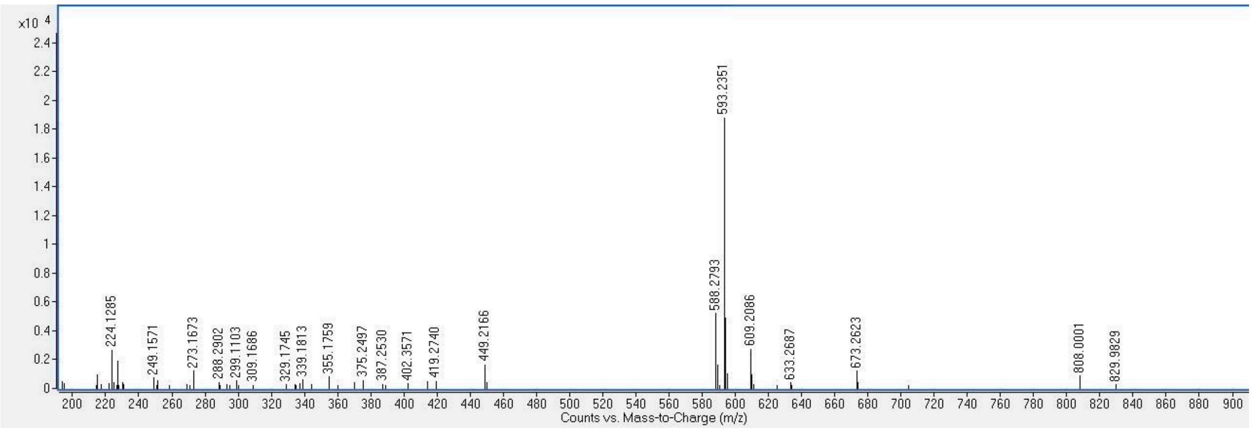

Figure S34. ESI (+) mass spectrum of component **35** eluting at t_R = 4.15 min in the chloroform extract of the latex of *E. cyparissias* (EC).

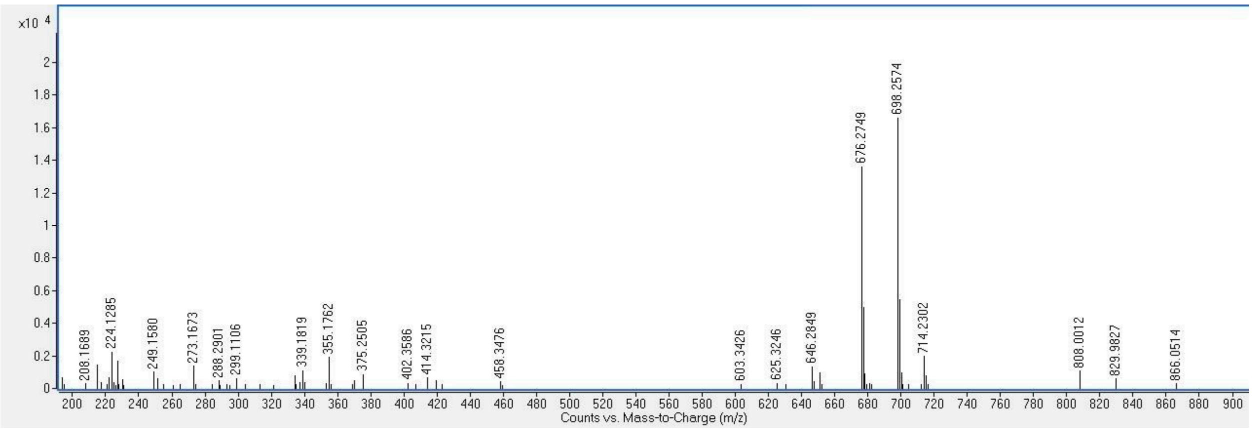

Figure S35. ESI (+) mass spectrum of component **36** eluting at t_R = 5.27 min in the chloroform extract of the latex of *E. cyparissias* (EC).

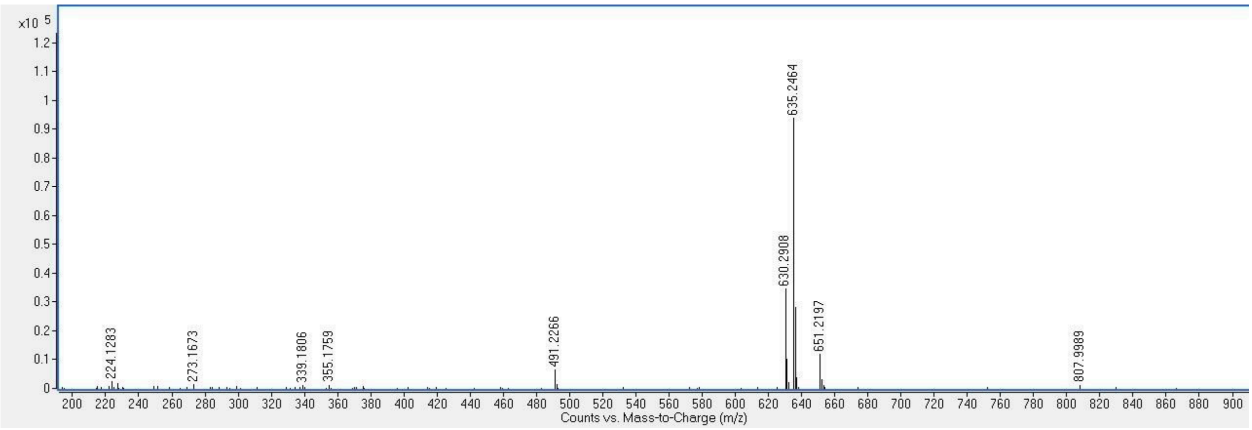

Figure S36. ESI (+) mass spectrum of component **37** eluting at t_R = 5.51 min in the chloroform extract of the latex of *E. cyparissias* (EC).

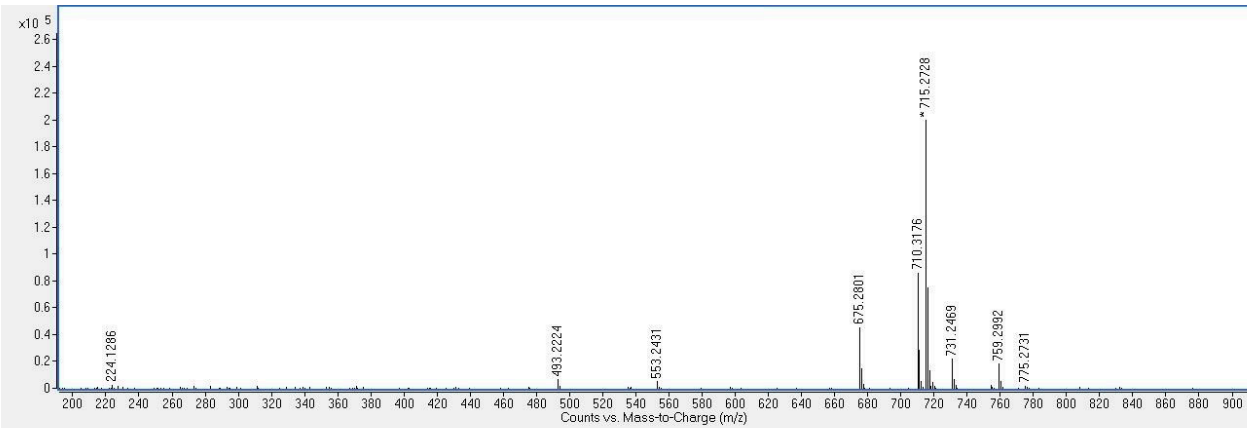

Figure S37. ESI (+) mass spectrum of component **38** eluting at t_R = 5.94 min in the chloroform extract of the latex of *E. cyparissias* (EC).

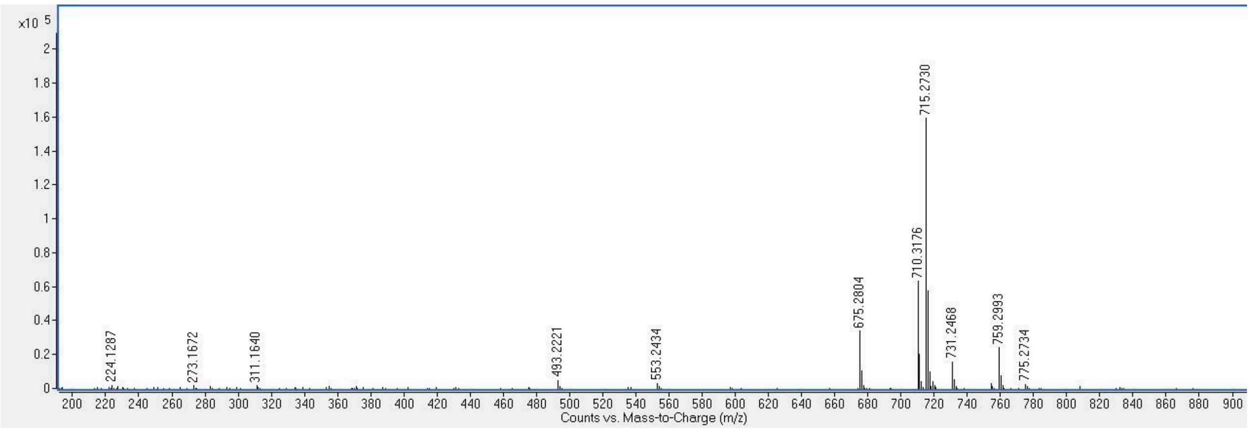

Figure S38. ESI (+) mass spectrum of component **39** eluting at t_R = 5.97 min in the chloroform extract of the latex of *E. cyparissias* (EC).

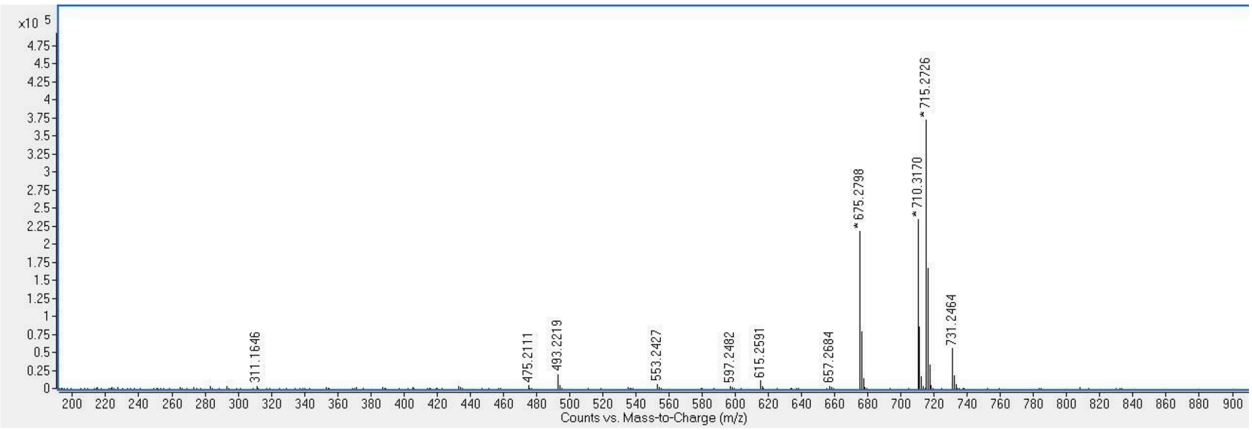

Figure S39. ESI (+) mass spectrum of component **40** eluting at t_R = 6.17 min in the chloroform extract of the latex of *E. cyparissias* (EC).

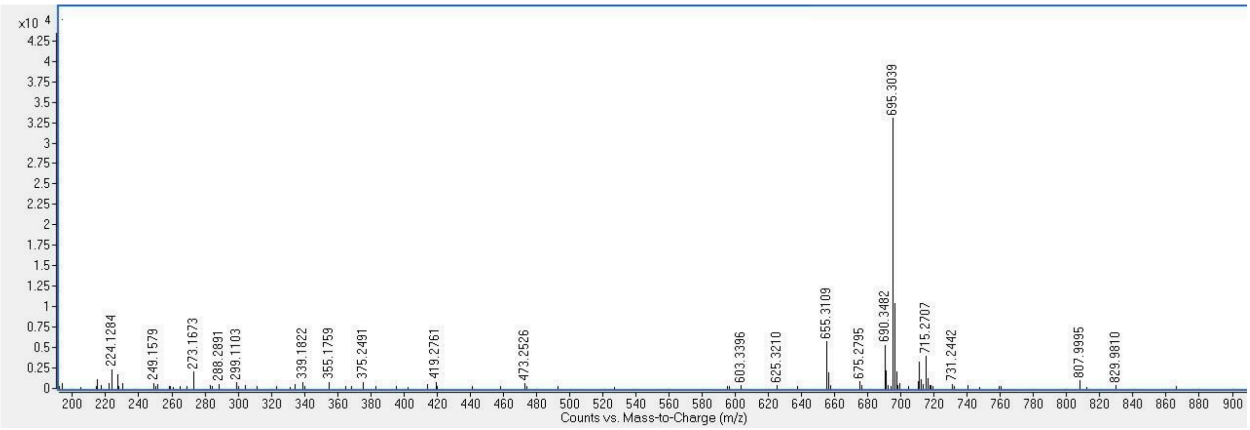

Figure S40. ESI (+) mass spectrum of component **41** eluting at t_R = 6.42 min in the chloroform extract of the latex of *E. cyparissias* (EC).

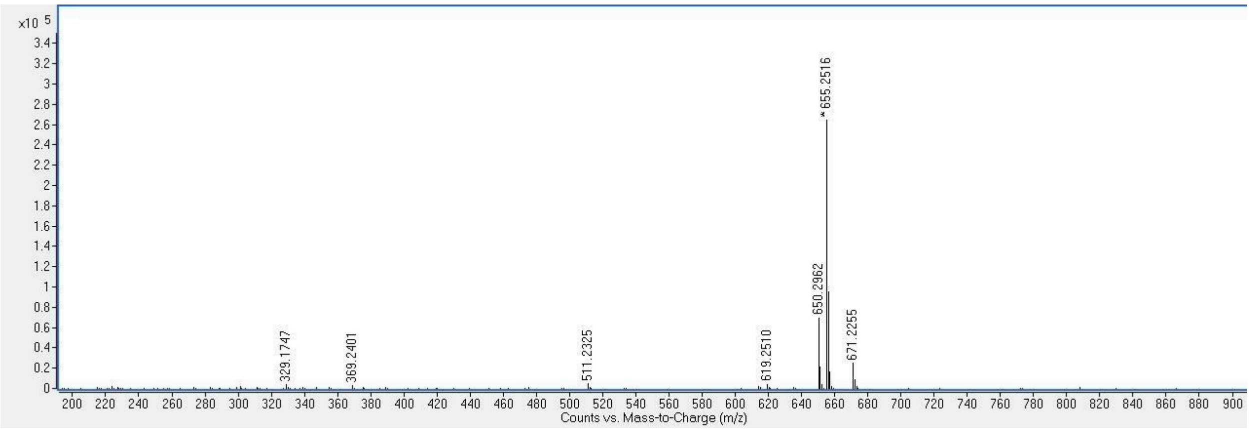

Figure S41. ESI (+) mass spectrum of component **42** eluting at t_R = 6.95 min in the chloroform extract of the latex of *E. cyparissias* (EC).

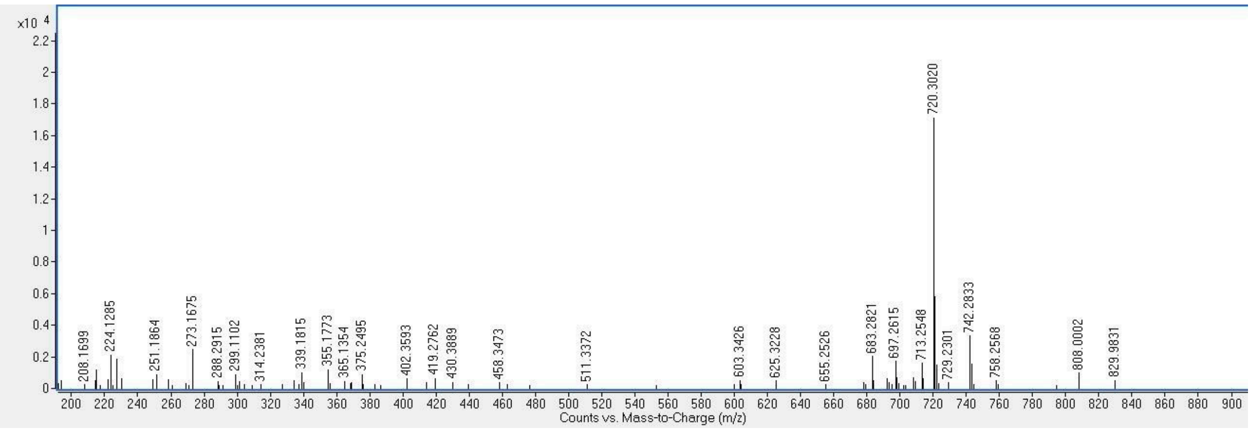

Figure S42. ESI (+) mass spectrum of component **43** eluting at t_R = 7.76 min in the chloroform extract of the latex of *E. cyparissias* (EC).

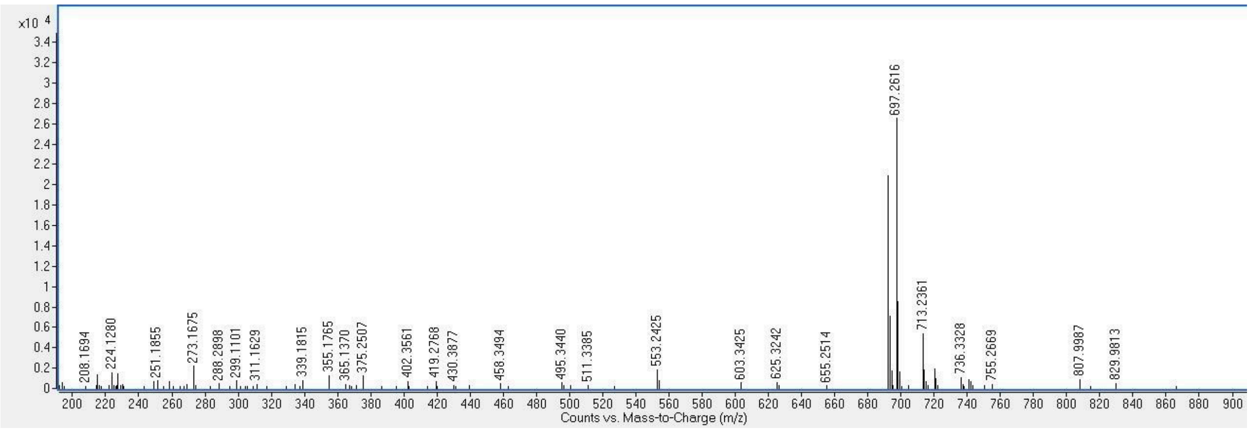

Figure S43. ESI (+) mass spectrum of component **44** eluting at t_R = 7.88 min in the chloroform extract of the latex of *E. cyparissias* (EC).

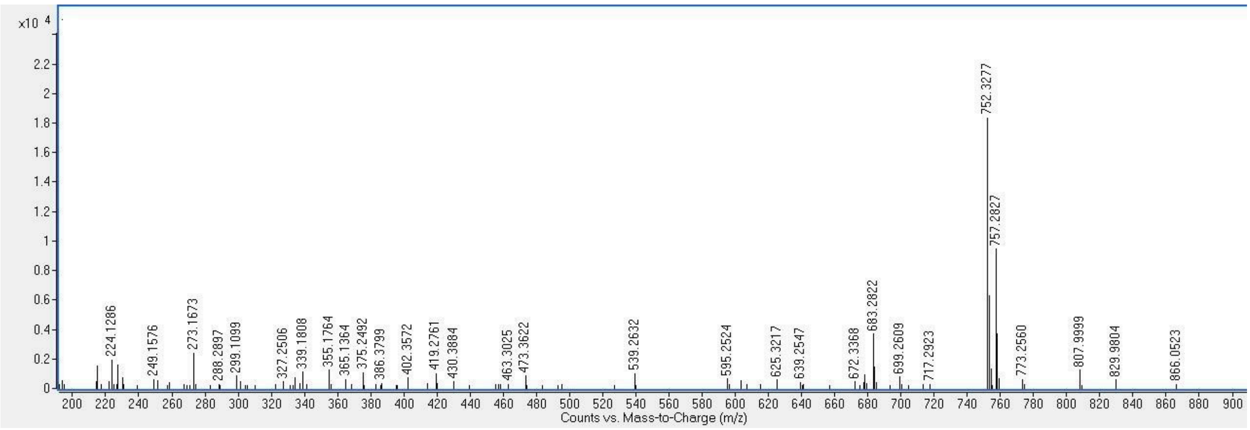

Figure S44. ESI (+) mass spectrum of component **45** eluting at t_R = 8.37 min in the chloroform extract of the latex of *E. cyparissias* (EC).

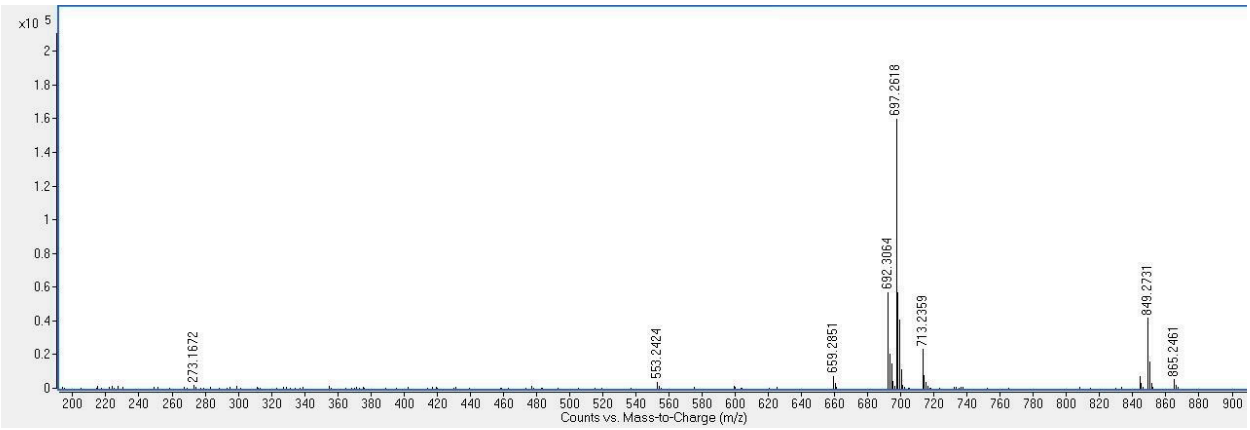

Figure S45. ESI (+) mass spectrum of component **46** eluting at t_R = 8.60 min in the chloroform extract of the latex of *E. cyparissias* (EC).

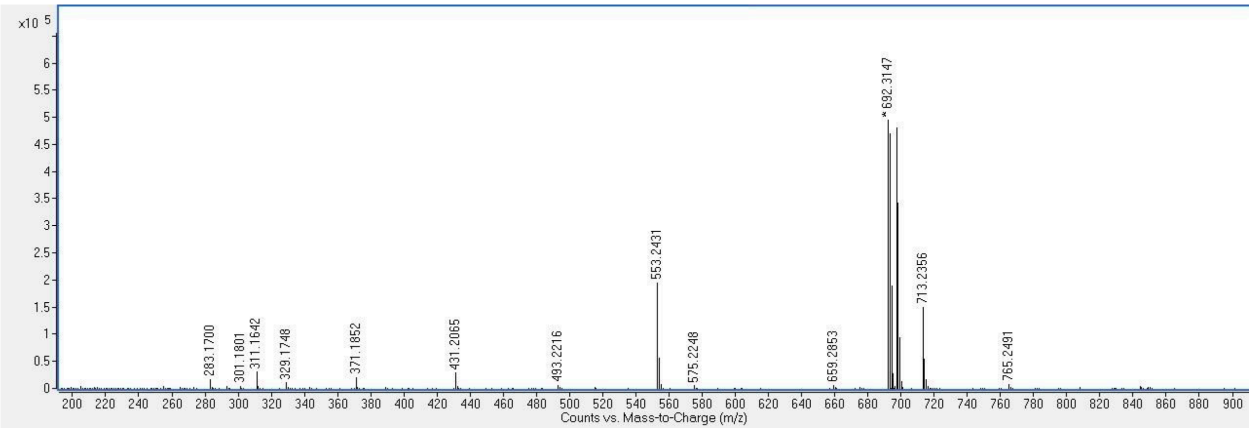

Figure S46. ESI (+) mass spectrum of component **47** eluting at t*R* = 8.70 min in the chloroform extract of the latex of *E. cyparissias* (EC).

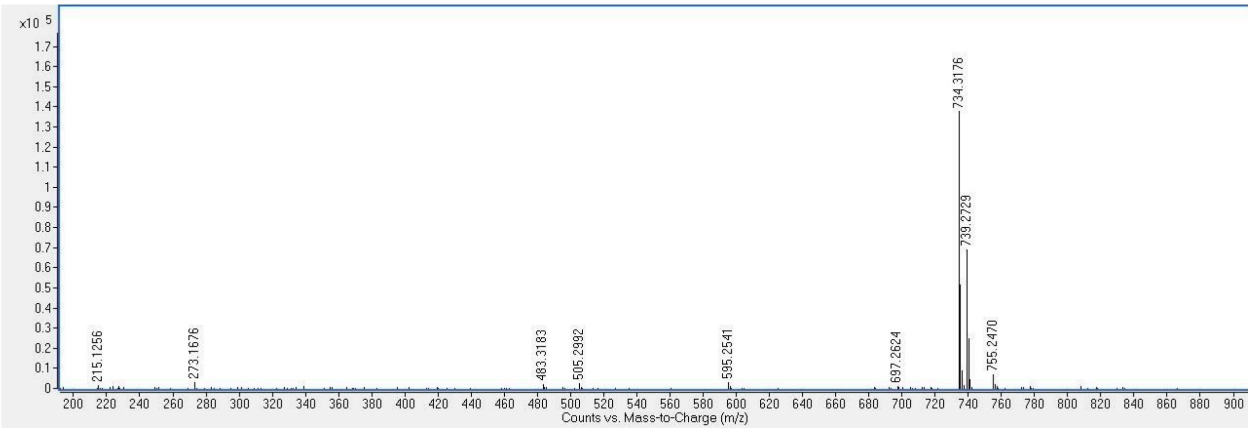

Figure S47. ESI (+) mass spectrum of component **48** eluting at t*R* = 9.26 min in the chloroform extract of the latex of *E. cyparissias* (EC).

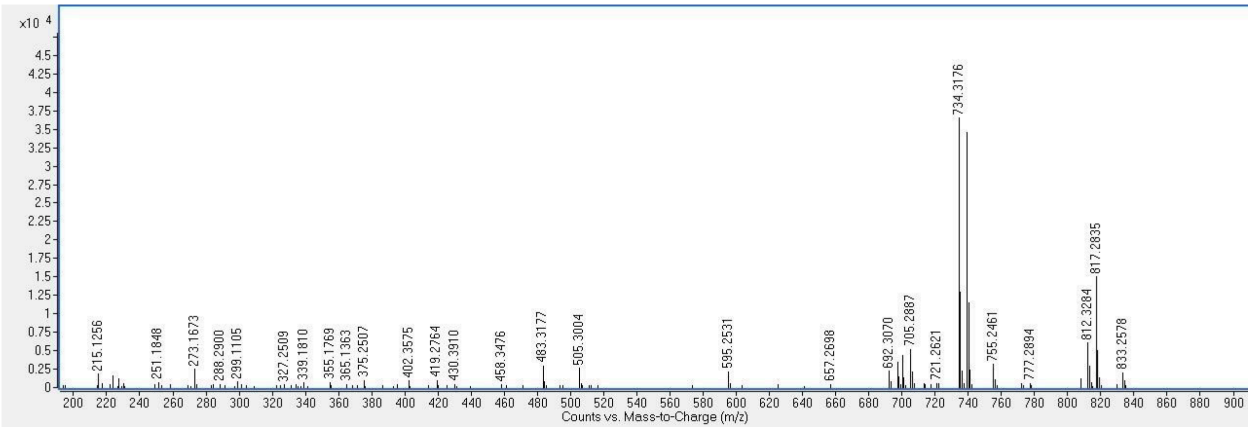

Figure S48. ESI (+) mass spectrum of component **49** eluting at t*R* = 9.36 min in the chloroform extract of the latex of *E. cyparissias* (EC).

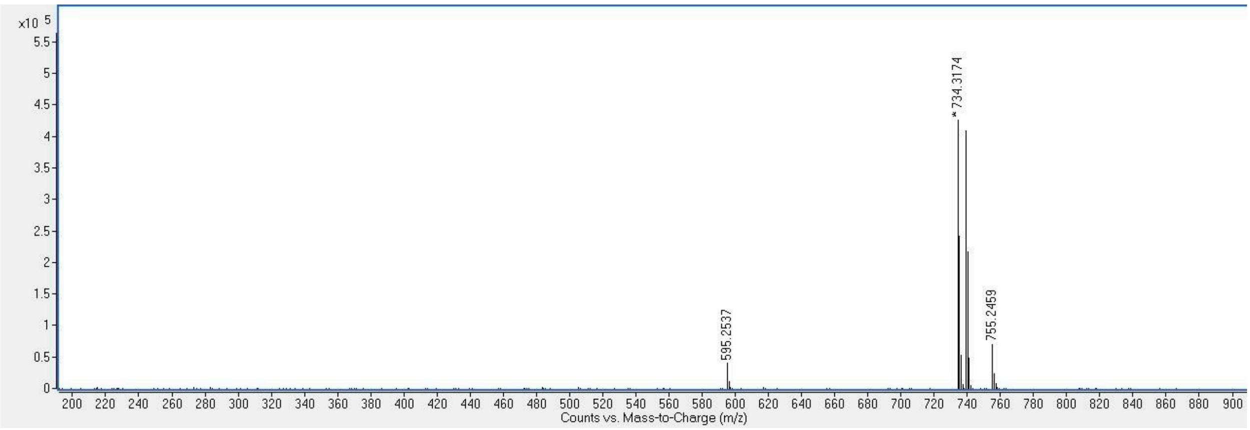

Figure S49. ESI (+) mass spectrum of component **50** eluting at t_R = 9.47 min in the chloroform extract of the latex of *E. cyparissias* (EC).

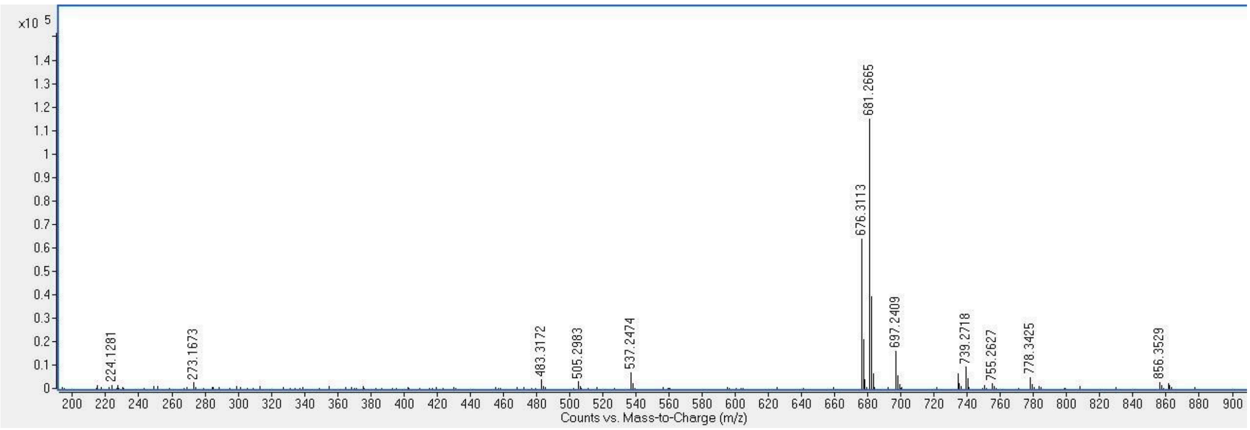

Figure S50. ESI (+) mass spectrum of component **51** eluting at t_R = 9.66 min in the chloroform extract of the latex of *E. cyparissias* (EC).

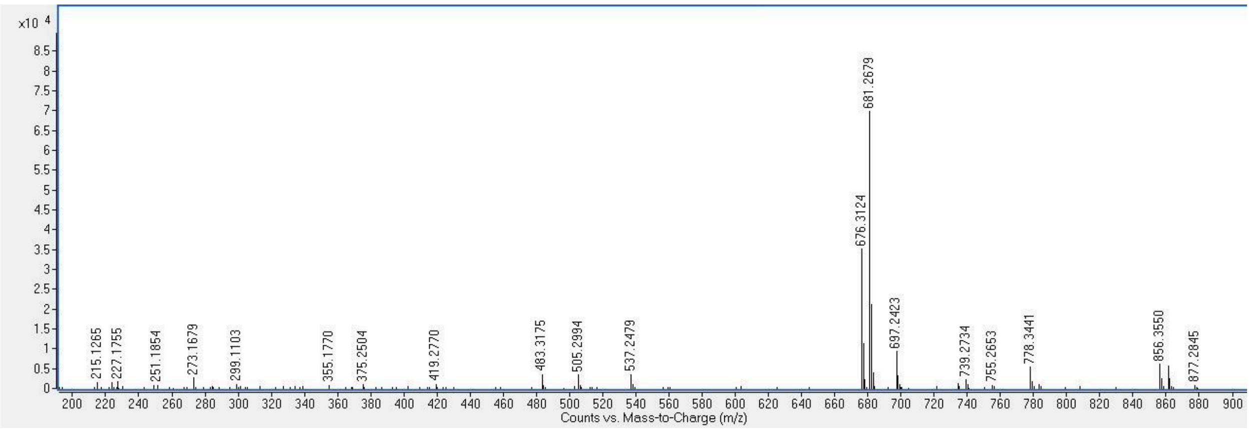

Figure S51. ESI (+) mass spectrum of component **52** eluting at t_R = 9.70 min in the chloroform extract of the latex of *E. cyparissias* (EC).

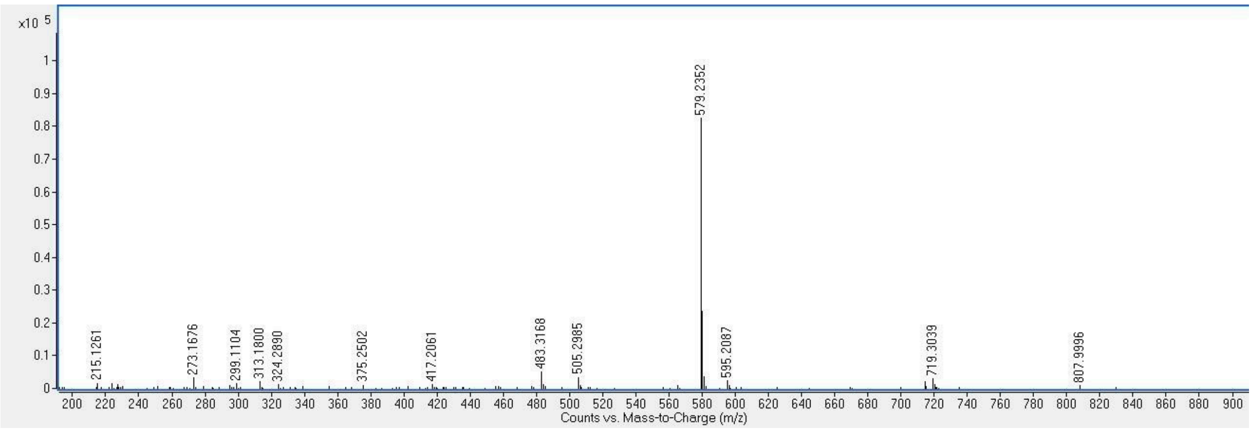

Figure S52. ESI (+) mass spectrum of component **53** eluting at t_R = 10.02 min in the chloroform extract of the latex of *E. cyparissias* (EC).

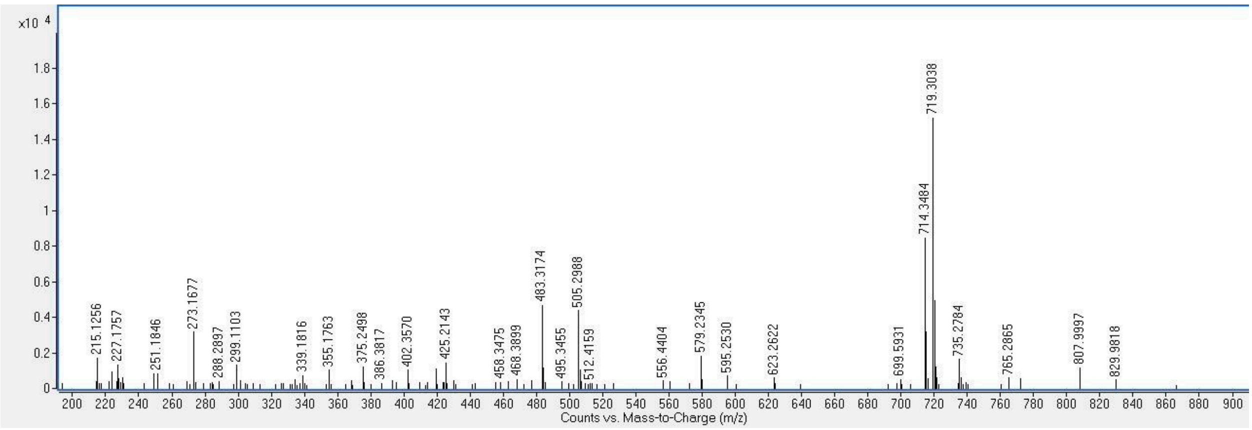

Figure S53. ESI (+) mass spectrum of component **54** eluting at t_R = 10.19 min in the chloroform extract of the latex of *E. cyparissias* (EC).

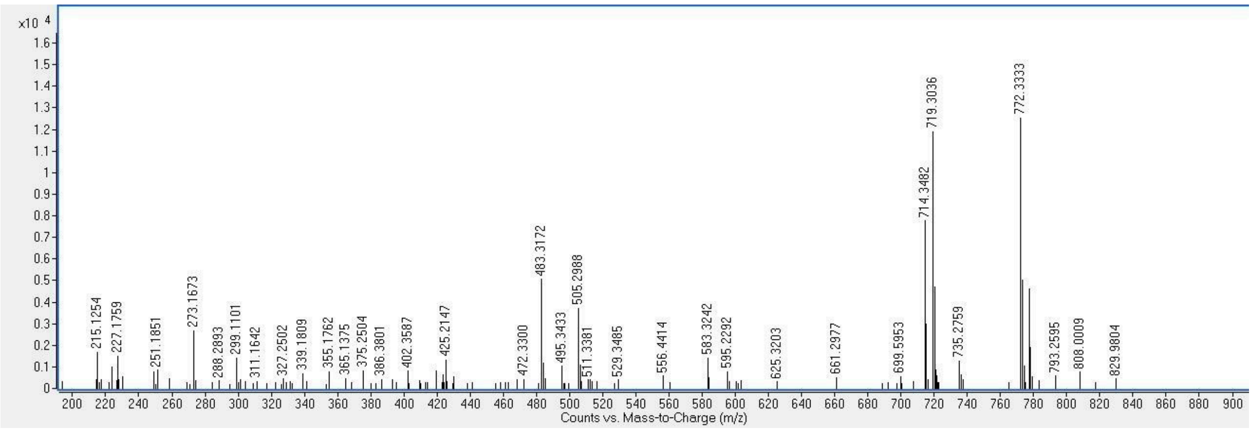

Figure S54. ESI (+) mass spectrum of component **55** eluting at t_R = 10.30 min in the chloroform extract of the latex of *E. cyparissias* (EC).

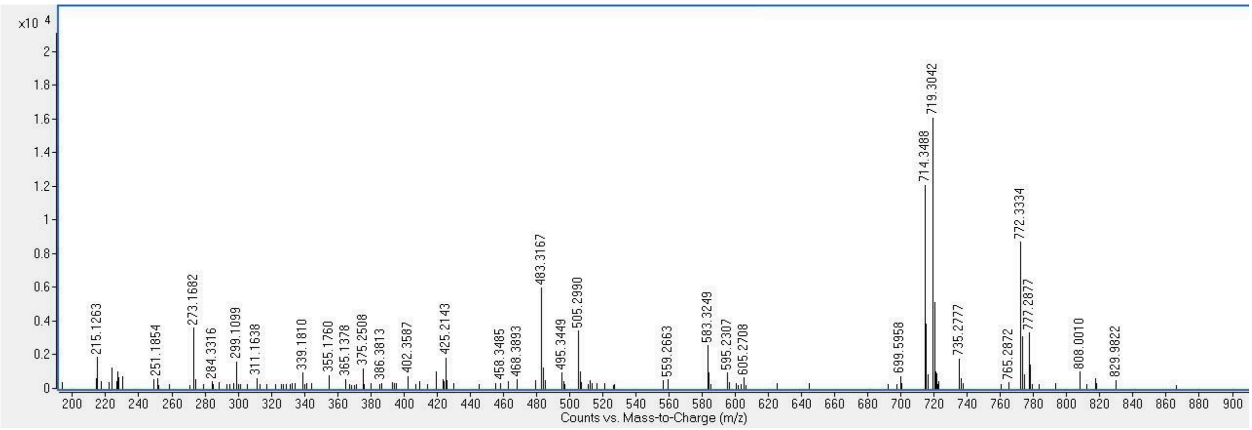

Figure S55. ESI (+) mass spectrum of component **56** eluting at t_R = 10.33 min in the chloroform extract of the latex of *E. cyparissias* (EC).

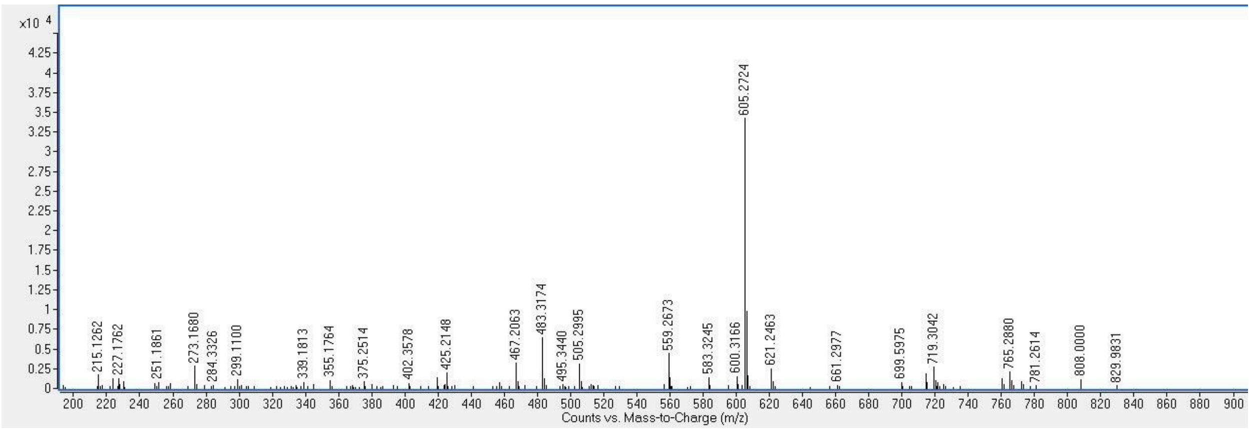

Figure S56. ESI (+) mass spectrum of component **57** eluting at t_R = 10.43 min in the chloroform extract of the latex of *E. cyparissias* (EC).

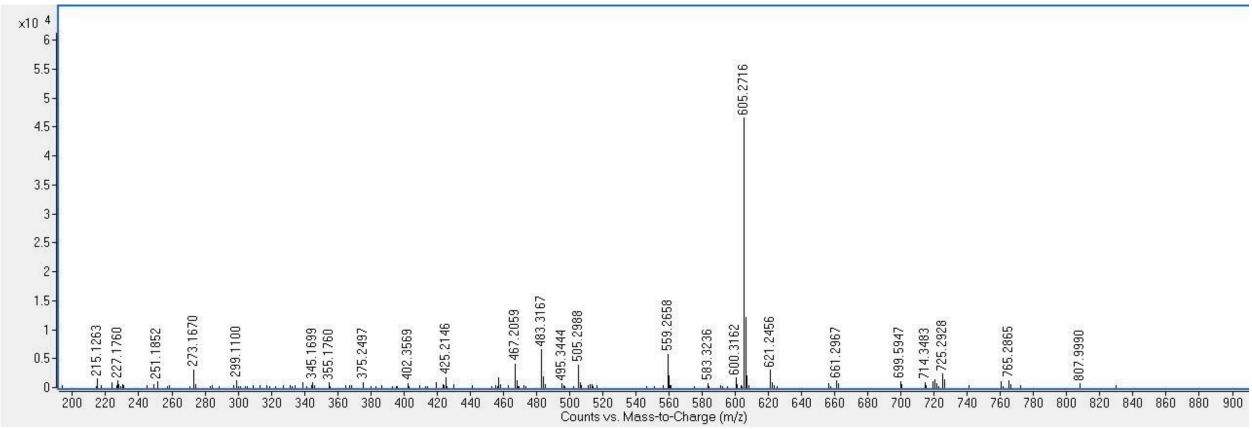

Figure S57. ESI (+) mass spectrum of component **58** eluting at t_R = 10.47 min in the chloroform extract of the latex of *E. cyparissias* (EC).

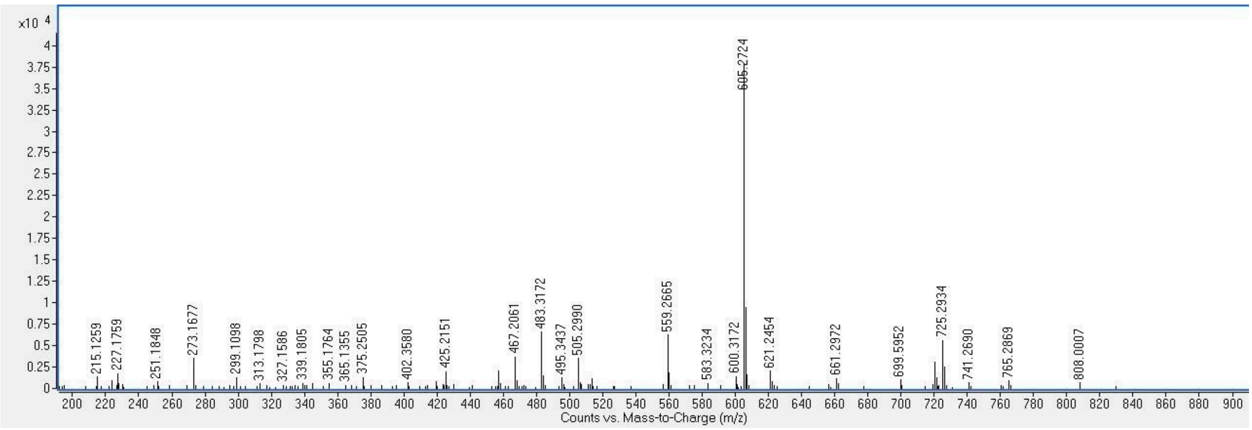

Figure S58. ESI (+) mass spectrum of component **59** eluting at t_R = 10.49 min in the chloroform extract of the latex of *E. cyparissias* (EC).

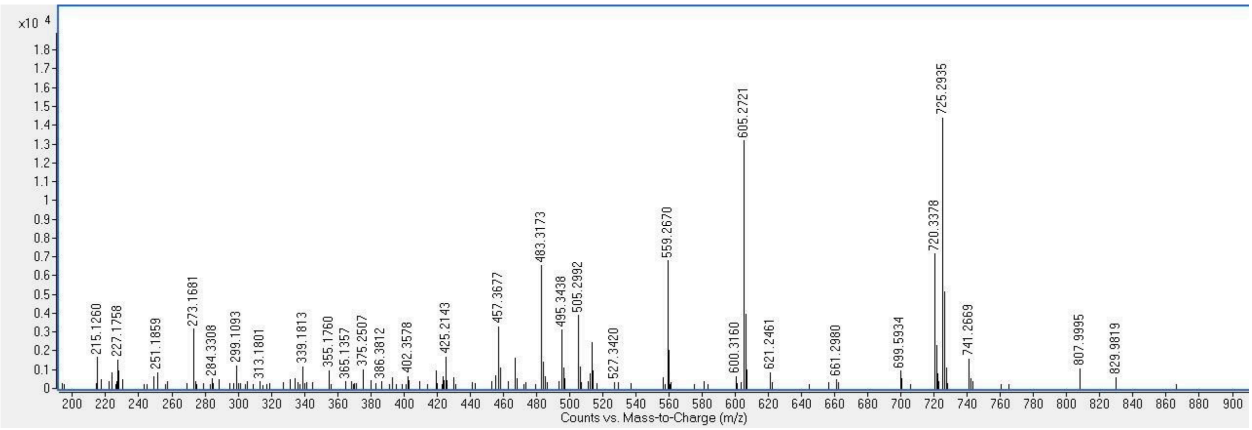

Figure S59. ESI (+) mass spectrum of component **60** eluting at t_R = 10.54 min in the chloroform extract of the latex of *E. cyparissias* (EC).

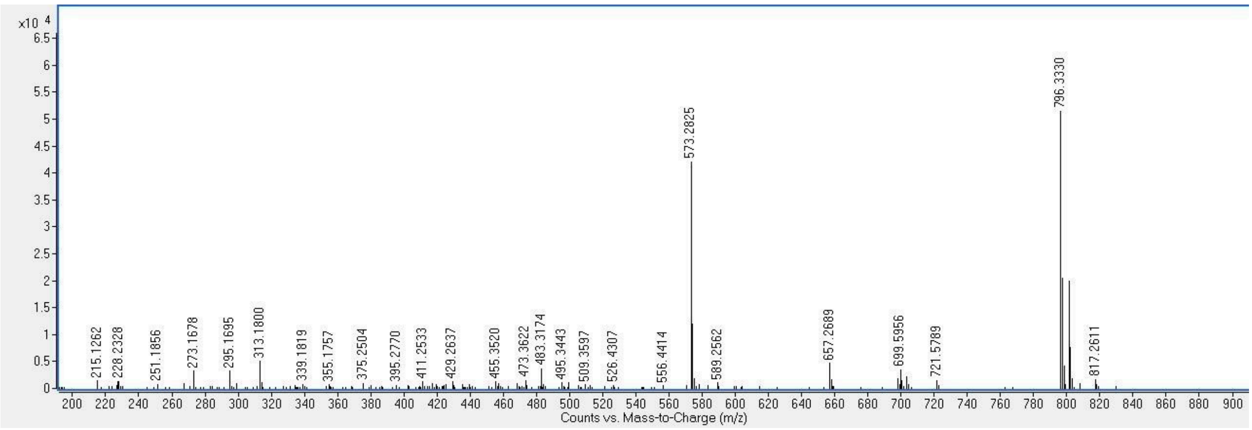

Figure S60. ESI (+) mass spectrum of component **61** eluting at t_R = 11.33 min in the chloroform extract of the latex of *E. cyparissias* (EC).

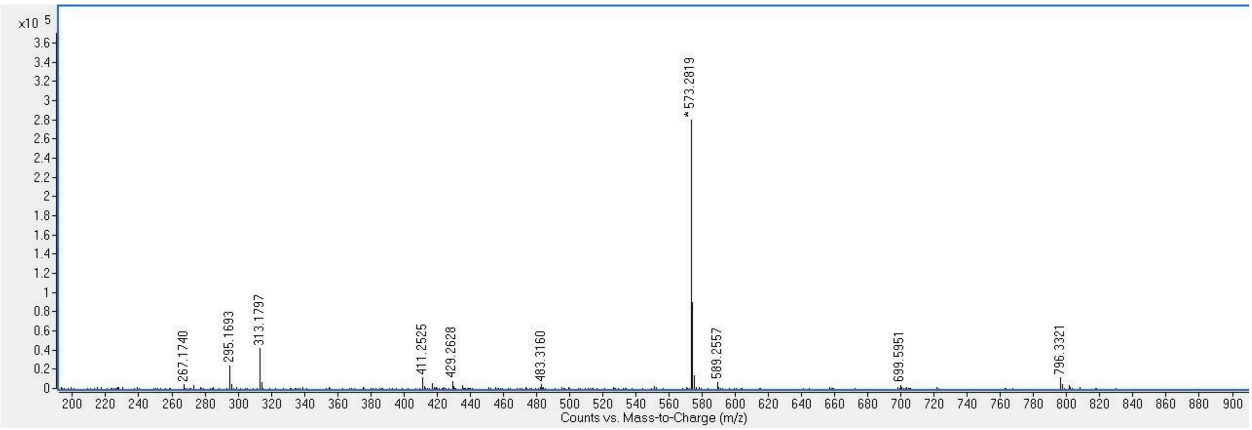

Figure S61. ESI (+) mass spectrum of component **62** eluting at t*R* = 11.41 min in the chloroform extract of the latex of *E. cyparissias* (EC).

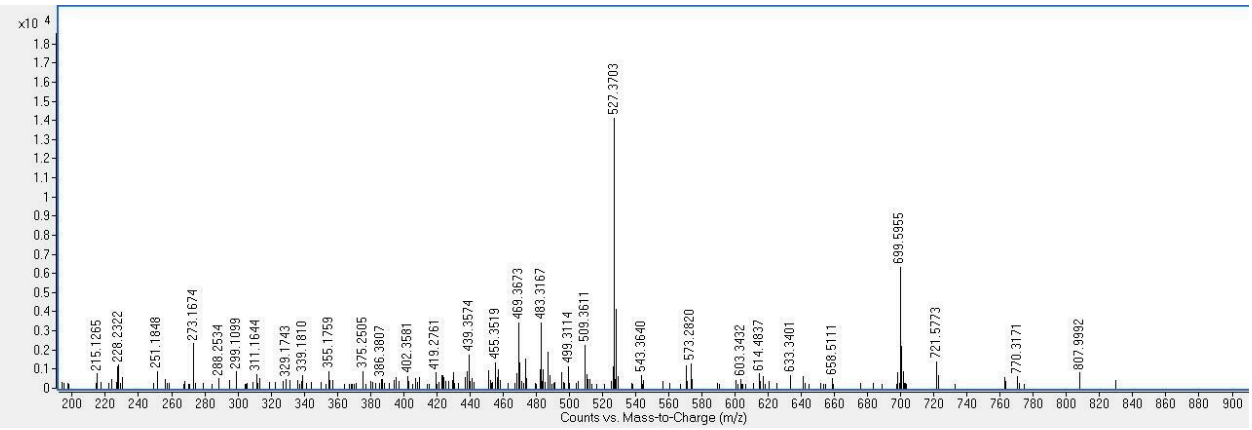

Figure S62. ESI (+) mass spectrum of component **63** eluting at t*R* = 11.64 min in the chloroform extract of the latex of *E. cyparissias* (EC).

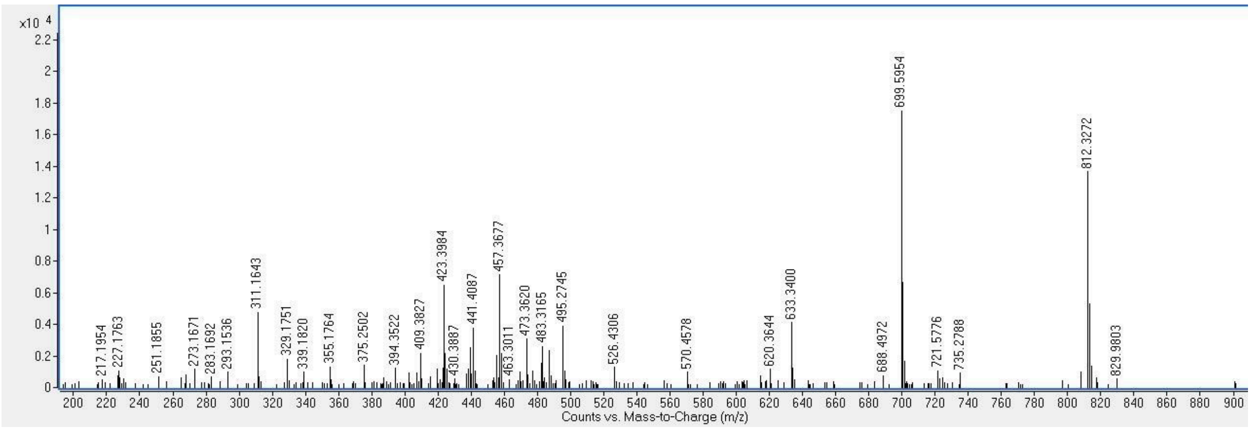

Figure S63. ESI (+) mass spectrum of component **64** eluting at t*R* = 12.51 min in the chloroform extract of the latex of *E. cyparissias* (EC).

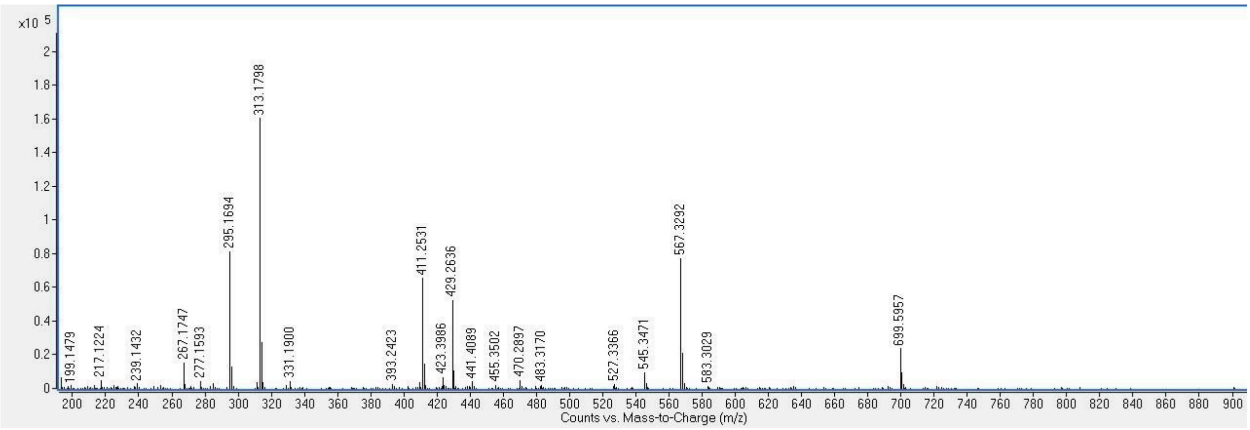

Figure S64. ESI (+) mass spectrum of component **65** eluting at t_R = 12.81 min in the chloroform extract of the latex of *E. cyparissias* (EC).

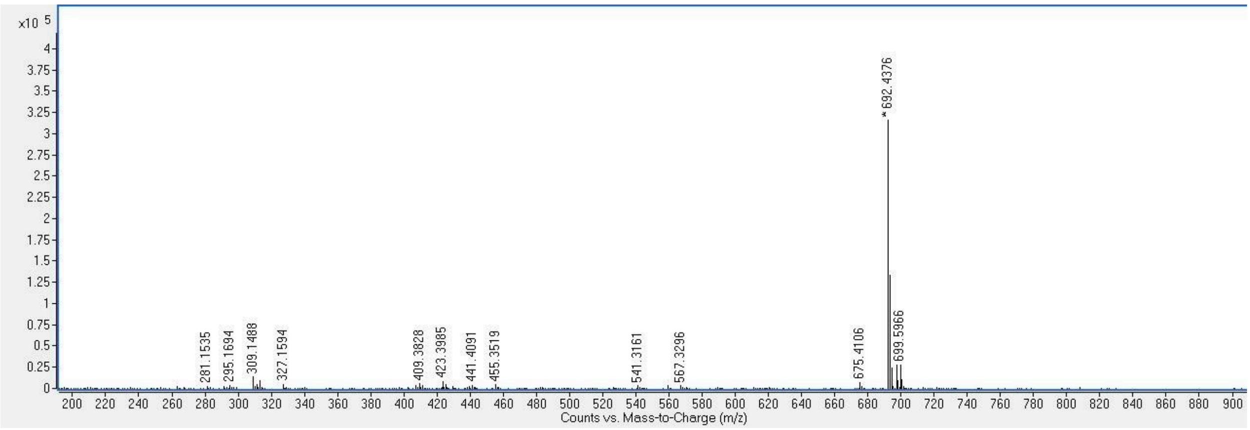

Figure S65. ESI (+) mass spectrum of component **66** eluting at t_R = 12.96 min in the chloroform extract of the latex of *E. cyparissias* (EC).

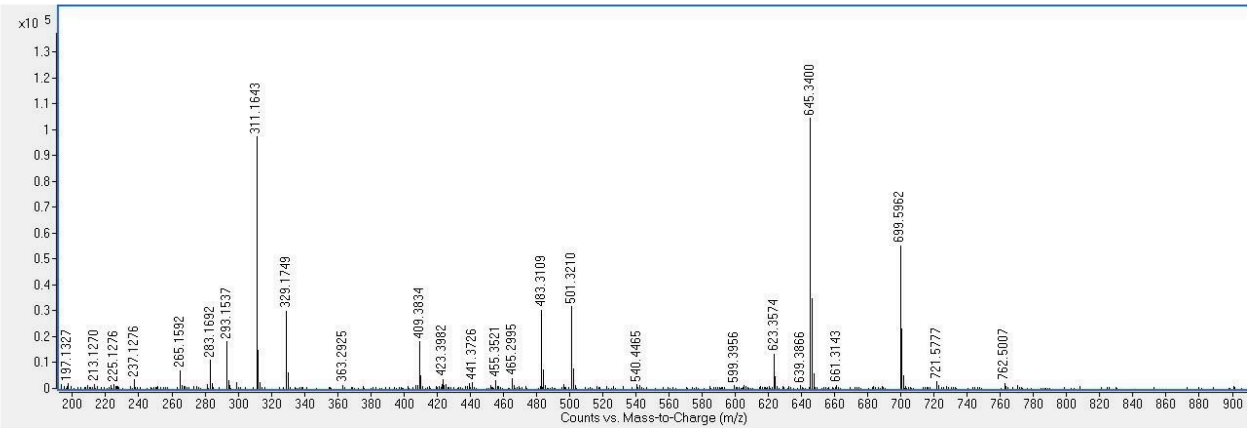

Figure S66. ESI (+) mass spectrum of component **67** eluting at t_R = 13.66 min in the chloroform extract of the latex of *E. cyparissias* (EC).

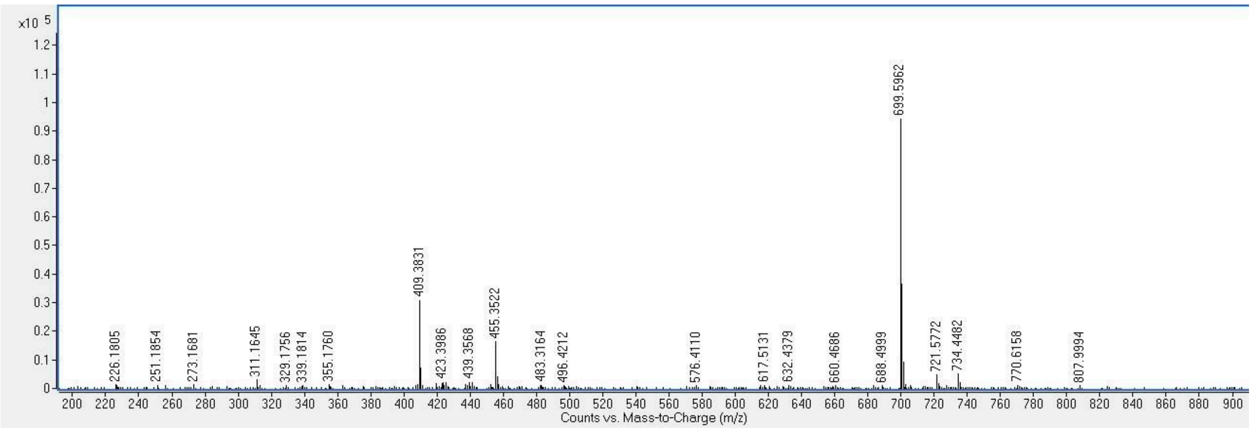

Figure S67. ESI (+) mass spectrum of component **68** eluting at t_R = 14.03 min in the chloroform extract of the latex of *E. cyparissias* (EC).

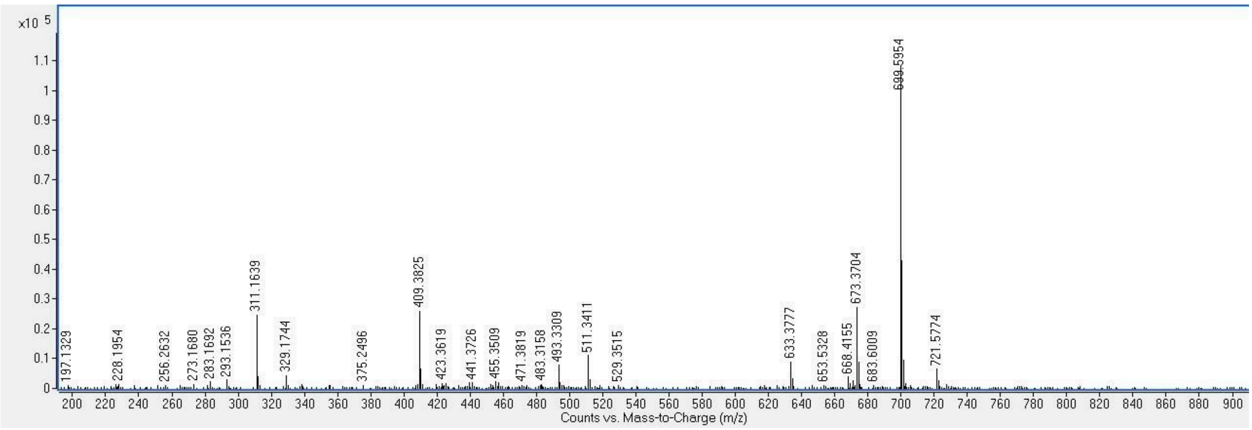

Figure S68. ESI (+) mass spectrum of component **69** eluting at t_R = 14.22 min in the chloroform extract of the latex of *E. cyparissias* (EC).

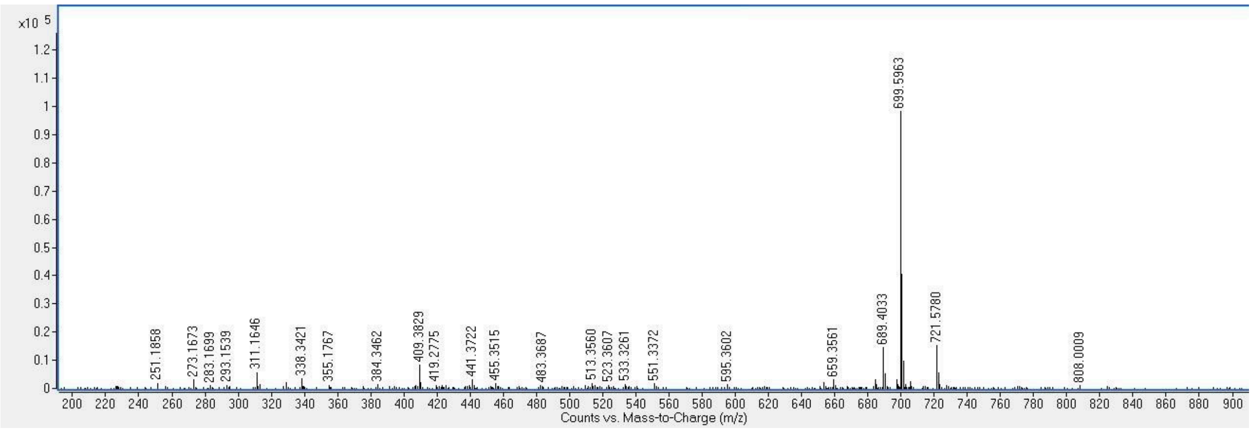

Figure S69. ESI (+) mass spectrum of component **70** eluting at t_R = 14.59 min in the chloroform extract of the latex of *E. cyparissias* (EC).

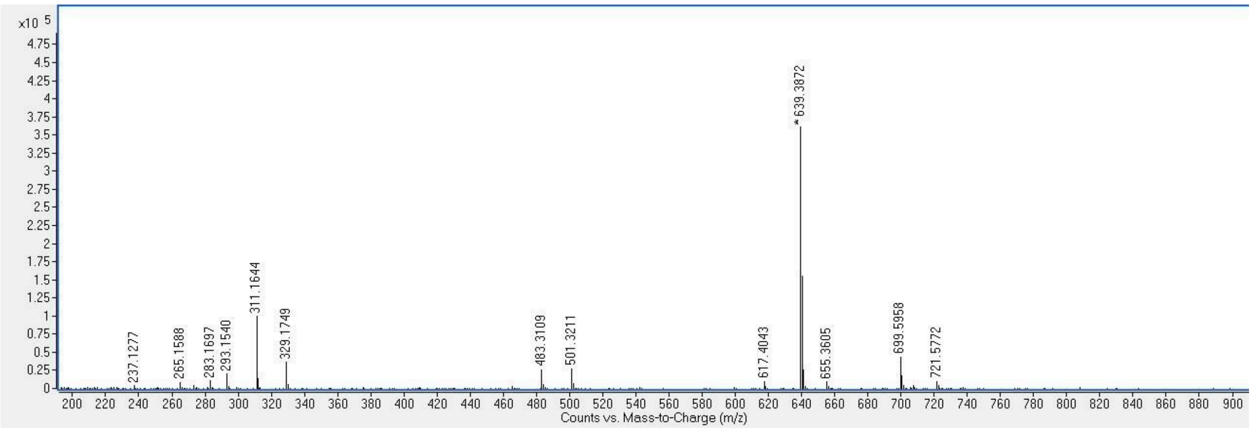

Figure S70. ESI (+) mass spectrum of component **71** eluting at t$_R$ = 14.98 min in the chloroform extract of the latex of *E. cyparissias* (EC).

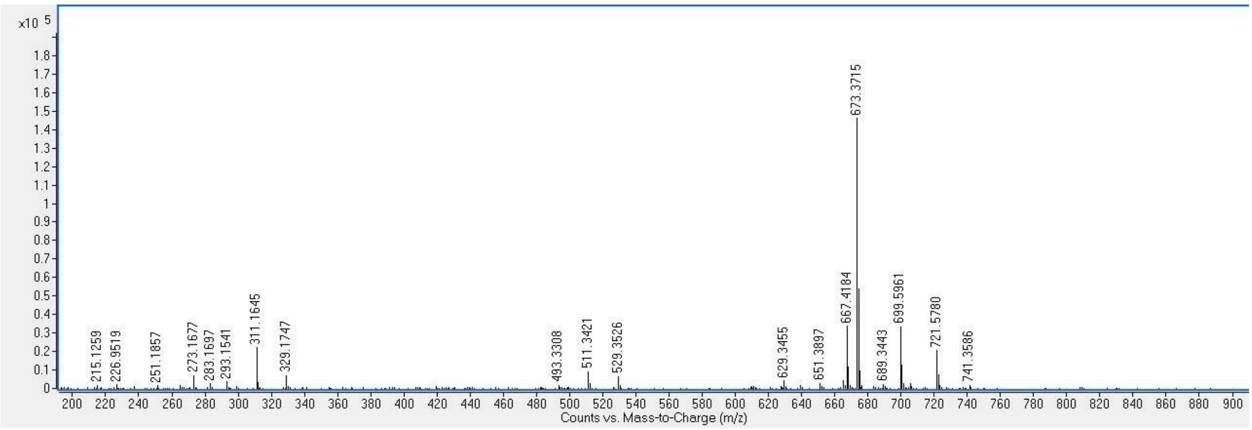

Figure S71. ESI (+) mass spectrum of component **72** eluting at t$_R$ = 15.63 min in the chloroform extract of the latex of *E. cyparissias* (EC).

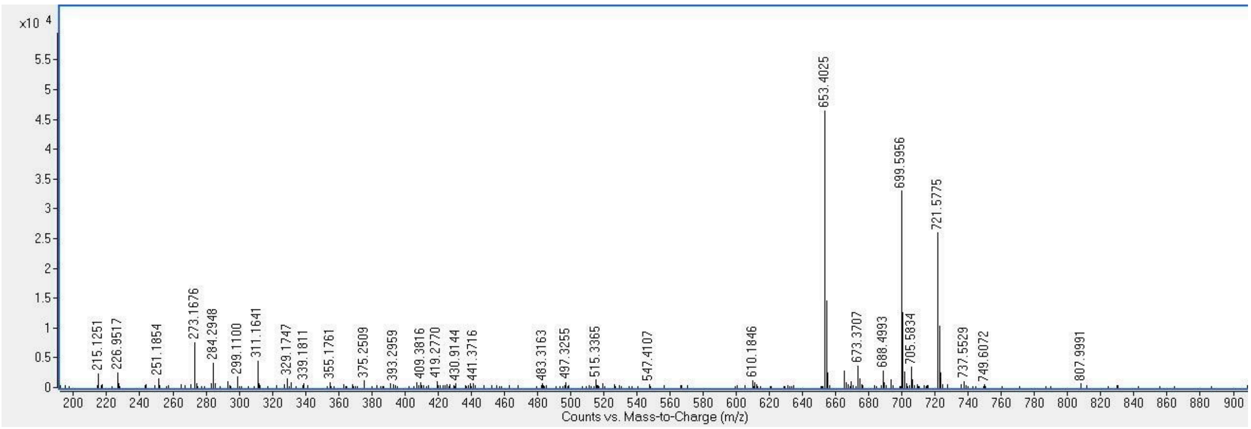

Figure S72. ESI (+) mass spectrum of component **73** eluting at t$_R$ = 15.84 min in the chloroform extract of the latex of *E. cyparissias* (EC).

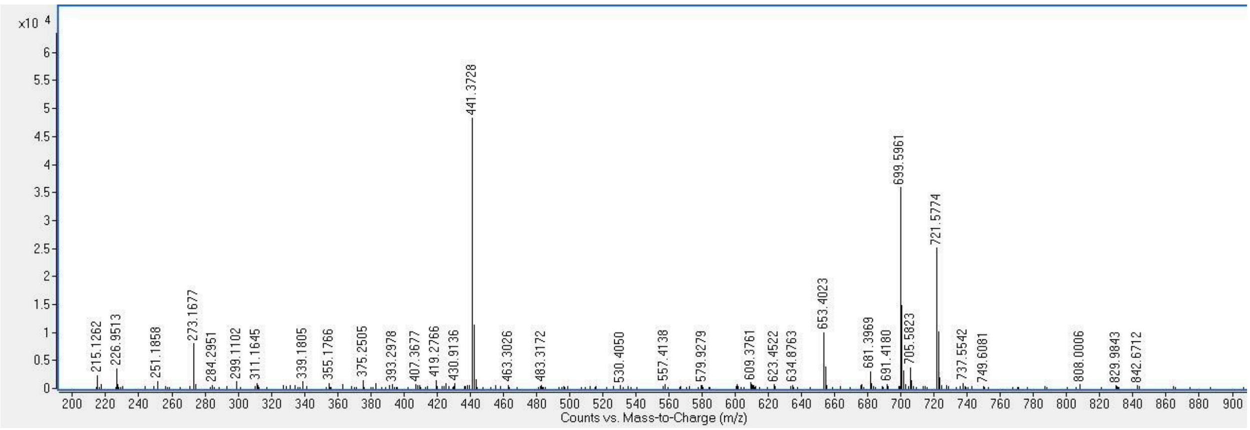

Figure S73. ESI (+) mass spectrum of component **74** eluting at t_R = 16.18 min in the chloroform extract of the latex of *E. cyparissias* (EC).

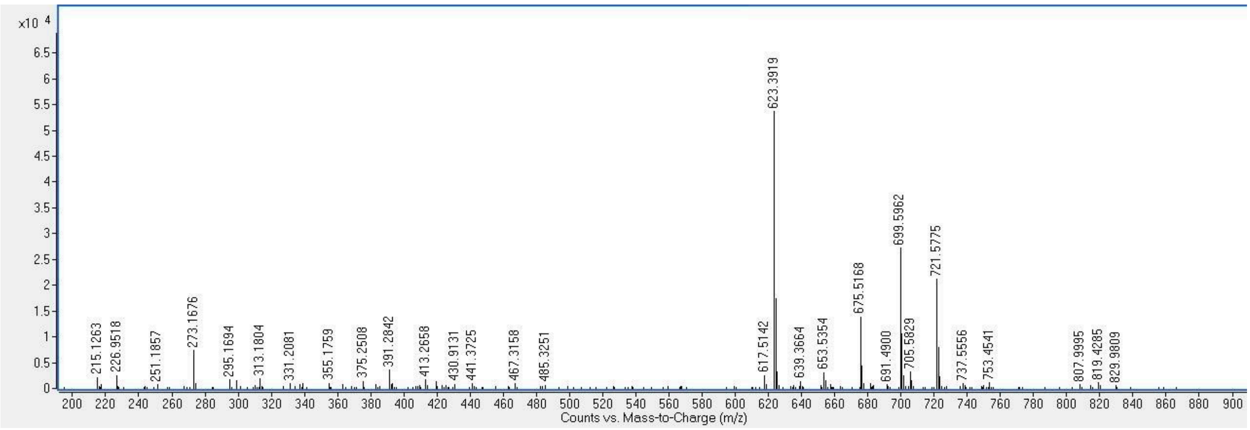

Figure S74. ESI (+) mass spectrum of component **75** eluting at t_R = 17.25 min in the chloroform extract of the latex of *E. cyparissias* (EC).

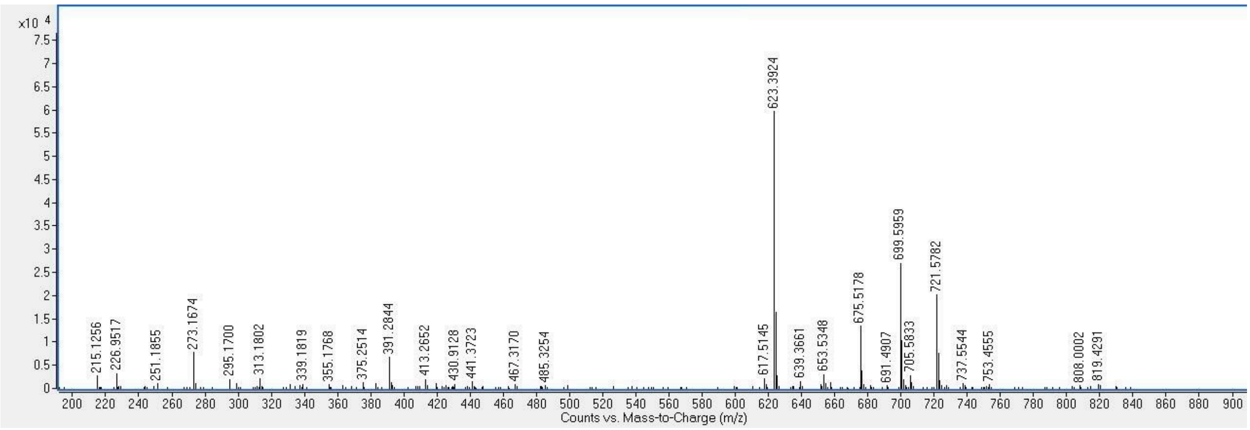

Figure S75. ESI (+) mass spectrum of component **76** eluting at t_R = 17.28 min in the chloroform extract of the latex of *E. cyparissias* (EC).

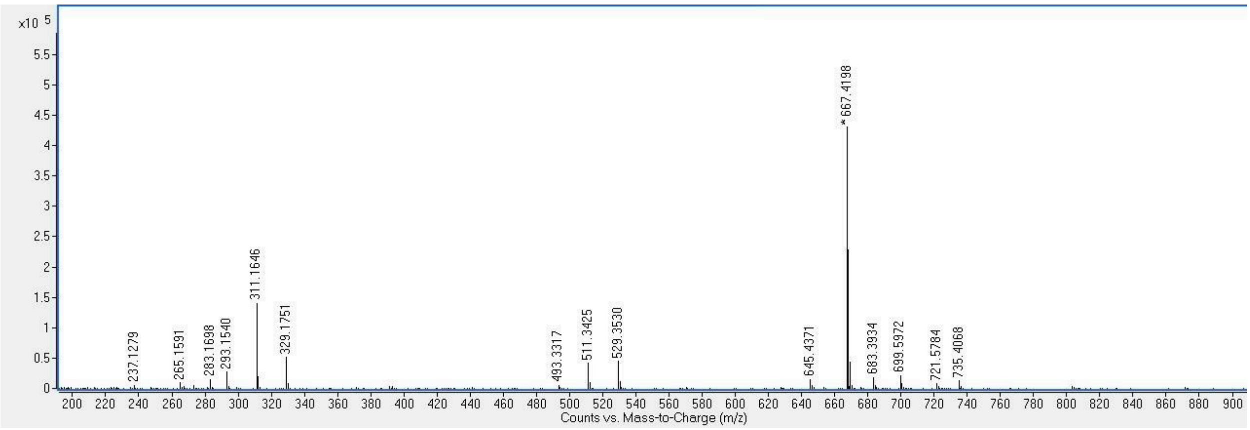

Figure S76. ESI (+) mass spectrum of component **77** eluting at t_R = 17.59 min in the chloroform extract of the latex of *E. cyparissias* (EC).

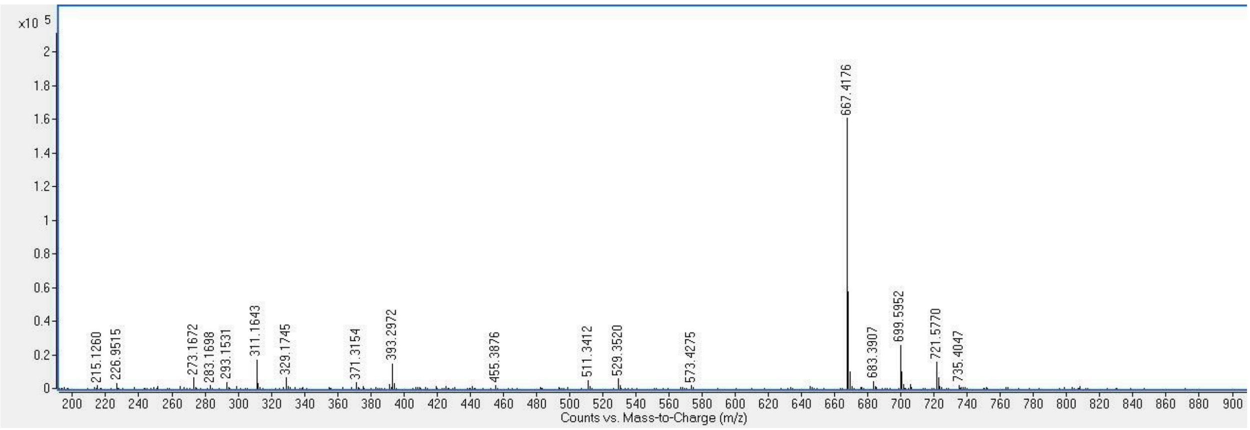

Figure S77. ESI (+) mass spectrum of component **78** eluting at t_R = 17.78 min in the chloroform extract of the latex of *E. cyparissias* (EC).

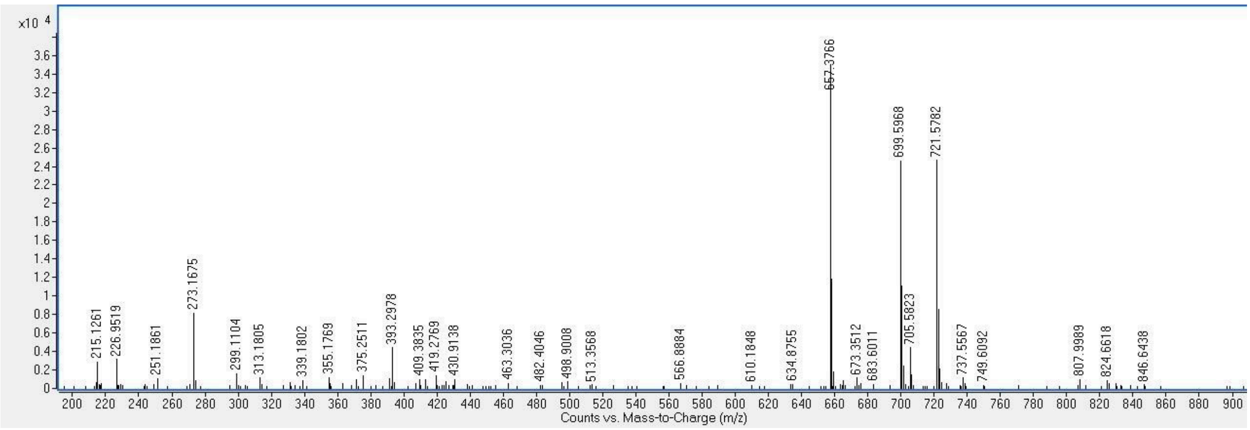

Figure S78. ESI (+) mass spectrum of component **79** eluting at t_R = 18.25 min in the chloroform extract of the latex of *E. cyparissias* (EC).

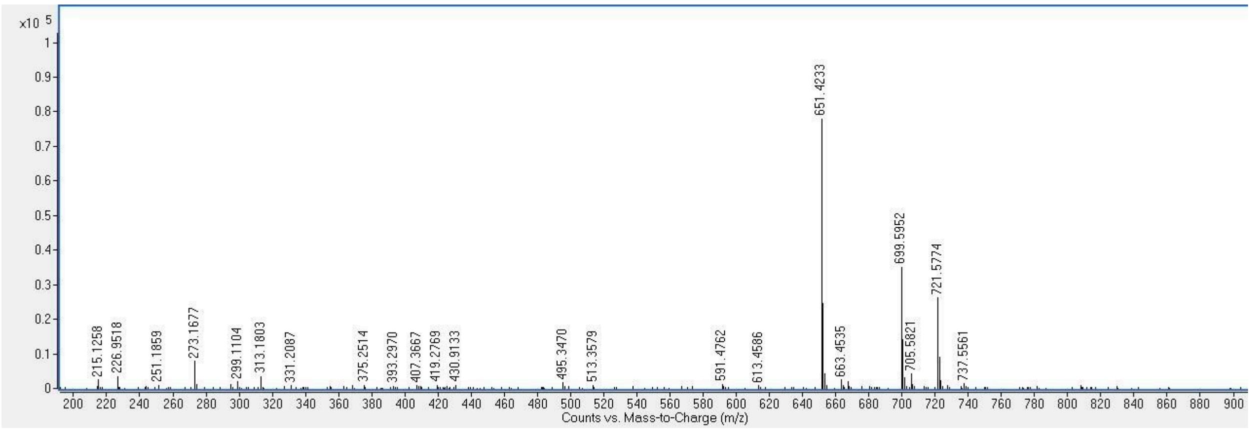

Figure S79. ESI (+) mass spectrum of component **80** eluting at t_R = 21.18 min in the chloroform extract of the latex of *E. cyparissias* (EC).
